# Multiple roles for the cytoplasmic C-terminal domains of the yeast cell surface receptors Rgt2 and Snf3 in glucose sensing and signaling

**DOI:** 10.1038/s41598-024-54628-2

**Published:** 2024-02-19

**Authors:** Jeong-Ho Kim, Levi Mailloux, Daniel Bloor, Haeun Tae, Han Nguyen, Morgan McDowell, Jaqueline Padilla, Anna DeWaard

**Affiliations:** Department of Biology and Chemistry, Liberty University, 1971 University Blvd, Lynchburg, VA 24502 USA

**Keywords:** Cell biology, Microbiology

## Abstract

The plasma membrane proteins Rgt2 and Snf3 are glucose sensing receptors (GSRs) that generate an intracellular signal for the induction of gene expression in response to high and low extracellular glucose concentrations, respectively. The GSRs consist of a 12-transmembrane glucose recognition domain and a cytoplasmic C-terminal signaling tail. The GSR tails are dissimilar in length and sequence, but their distinct roles in glucose signal transduction are poorly understood. Here, we show that swapping the tails between Rgt2 and Snf3 does not alter the signaling activity of the GSRs, so long as their tails are phosphorylated in a Yck-dependent manner. Attachment of the GSR tails to Hxt1 converts the transporter into a glucose receptor; however, the tails attached to Hxt1 are not phosphorylated by the Ycks, resulting in only partial signaling. Moreover, in response to non-fermentable carbon substrates, Rgt2 and Hxt1-RT (RT, Rgt2-tail) are efficiently endocytosed, whereas Snf3 and Hxt1-ST (ST, Snf3-tail) are endocytosis-impaired. Thus, the tails are important regulatory domains required for the endocytosis of the Rgt2 and Snf3 glucose sensing receptors triggered by different cellular stimuli. Taken together, these results suggest multiple roles for the tail domains in GSR-mediated glucose sensing and signaling.

## Introduction

Yeast can metabolize glucose efficiently over a broad range of concentrations from a few micromolar to a few molar, and accordingly, they have evolved sophisticated mechanisms for sensing the amount of glucose available and responding appropriately^1^. The budding yeast *S. cerevisiae* senses extracellular glucose levels through the glucose transporter-like proteins Rgt2 and Snf3^2,3^. However, these proteins are unable to transport glucose but do generate an intracellular signal that leads to expression of the genes involved in glucose transport and metabolism, including the glucose transporter (*HXT*) genes^4^. Signal generation is receptor-mediated because glucose metabolism is not necessary for its generation, and furthermore, there are dominant mutations in the *RGT2* and *SNF3* genes that cause constitutive signal generation^5^. These observations have led to the view that these proteins are metabolism-independent glucose sensing receptors (GSRs), formerly known as glucose sensors^1,5^.

The GSR protein levels are regulated by different mechanisms—glucose starvation-induced endocytic degradation of the Rgt2 receptor and high glucose-induced repression of *SNF3* gene expression^6^. As a result, Rgt2 is more prevalent than Snf3 in cells grown in high glucose, whereas Snf3 is more abundant than Rgt2 when extracellular glucose levels are low^6^. When expressed from a constitutive promoter, however, Snf3 behaves like Rgt2, being able to generate a signal in response to high glucose that induces high-glucose-induced *HXT1* expression. Thus, the distinct roles of Rgt2 and Snf3 in high- and low-glucose-induced gene expression may be due to their different expression levels at various concentrations of extracellular glucose, rather than a result of the two paralogous proteins having different functions. Furthermore, the *RGT2-1* and *SNF3-1* dominant mutants both exhibit constitutive expression of both the high- and low-glucose-induced genes^5^, suggesting that Rgt2 and Snf3 generate the same signal that induces *HXT* expression.

The GSR downstream signaling pathway has been well characterized^1,7,8^. However, it is unclear how the signal is generated by the GSRs and transmitted to the intracellular GSR signaling pathway. Previous studies suggested that glucose binding to the GSRs would activate the plasma membrane (PM)-tethered casein kinases Yck1 and Yck2 (CKI), the human homologs of the casein kinase 1-gamma (CK1γ), leading to the phosphorylation and degradation of the *HXT* corepressors Mth1 and Std1 and the consequent release of the Rgt1 repressor from the *HXT* promoters^9–12^. Thus, the glucose signal induces *HXT* gene expression by ultimately effecting the dissociation of Rgt1 from the *HXT* promoters that results in *HXT* expression^13,14^. However, recent studies provide evidence that the GSRs are epistatic to the Ycks, placing the kinases upstream or at the level of the GSRs in the glucose signaling pathway^15,16^, and that the catalytic activity of the Ycks is not regulated by the GSRs but is constitutively active^17^. Moreover, the Ycks appear to be required for the stability and function of the Rgt2 receptor^17^. These results suggest that the Ycks may be upstream regulators of the GSRs and implicate their role in the generation of a glucose signal by the GSRs.

The Rgt2 and Snf3 GSRs are composed of two functionally distinct domains; the 12 transmembrane (TM) input domains that contain a glucose binding site and an unusually long C-terminal cytoplasmic output domain (CTD or “tail”) (213 and 337 amino acids in Rgt2 and Snf3, respectively), which serves as a signaling domain^3,18,19^. The attachment of the tails of GSRs to the Hxt1 and Hxt2 glucose transporters converts them into glucose receptors that can then generate an intracellular signal for induction of *HXT* expression^4,9^. Interestingly, a tail-less Rgt2 appears to generate the glucose signal when overexpressed, suggesting that the tails are not required for signal generation but serve to enhance signaling^9^. However, early studies suggested that the CTD of the Snf3 receptor is sufficient to complement the growth defect of *snf3* null mutations^19^, and that the Snf3-CTD, when expressed independently of the TM domains, leads to glucose-independent expression of *HXT2* on gluconeogenic carbon sources^20^. Furthermore, the Snf3 tail exhibits a signaling activity that is independent of the presence of extracellular glucose^21^.

In this study, we explored the roles of the GSR tails in glucose sensing and signaling using chimeric GSRs, hybrid receptor/transporter proteins, mutagenesis, and homology modeling. Our results revealed that the tails not only play an important role in transmitting the signal generated by the TM domains but also have multiple roles in the GSR-mediated glucose-dependent and -independent signaling.

## Results

### The C-terminal tails are required for endocytic degradation of the GSRs

**Figure 1:**
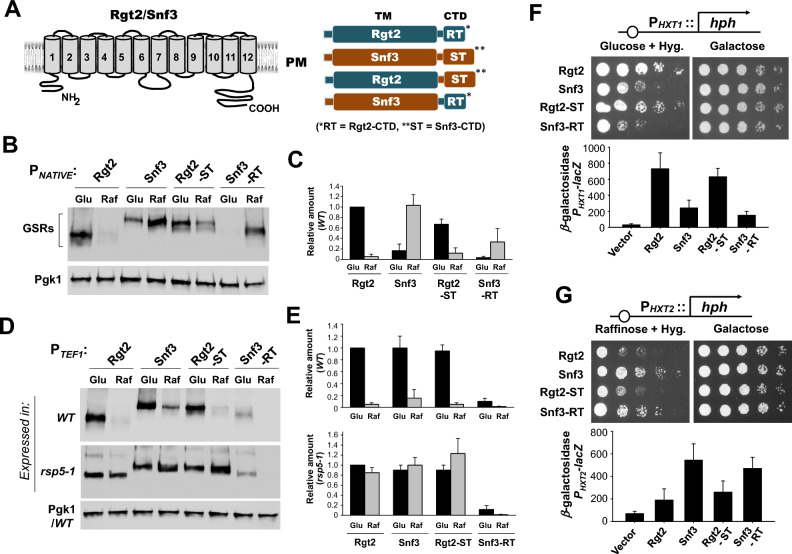
Swapping the tails between Rgt2 and Snf3 does not alter the signaling specificity of the GSRs. (**A**) The predicted transmembrane topology model of the Rgt2 and Snf3 GSRs (left) and the schematic representation of the structures of the native and chimeric receptors (right). RT and ST represent the tail domains of Rgt2 and Snf3, respectively. (**B**) Western blot analysis of protein levels of the wild-type and chimeric GSRs. C-terminally HA-tagged GSRs (*RGT2-HA* (JKP253, 763aa + 3HA), *SNF3-HA* (JKP298, 884aa + 3HA), *RGT-ST* (JKP602, 887aa + 3HA), and *SNF3-RT* (JKP600, 760aa + 3HA)) were expressed from their native promoters. Wild-type cells (BY4742)^52^ were grown in selective SC medium with 2% glucose to mid-log phase (O.D_600nm_ = 1.2–1.5) and equal amounts of cells were shifted to SC medium containing glucose (2%) or raffinose (2%) for 1 h. Membrane fractions were immunoblotted with anti-HA antibody. Pgk1 was used as loading control. (**C**) Relative band intensities (**B**) were quantified densitometrically using the NIH ImageJ program^17^. Averages of glucose samples (Rgt2-HA) were set to 1. Results were obtained from at least three independent experiments. (**D**) Western blot analysis of the wild-type and chimeric GSRs expressed from the constitutive *TEF1* promoter in wild-type^53^ and *rsp5-1* cells^53^. PGK1 was expressed from the wild type cells. Cells were grown and treated as described above (B). The *GRS* genes expressed were: *RGT2* (JKP443), *SNF3* (JKP444), *RGT2-ST* (JKP609), and *SNF3-RT* (JKP631). (**E**) Relative band intensities (**D**) were quantified as described above (**C**). (**F**) The P_*HXT1*_*-hph* reporter strains (KLS76)^6^ expressing the indicated *GSR* genes were scored for growth in a SC-2% glucose plate supplemented with 200 µg/ml hygromycin or galactose (2%) plate. The first spot of each row represents a count of ~ 5 × 10^7^ cell/ml, which is diluted 1:10 for each spot thereafter (top). The plates were incubated for 2 days at 30 °C. The *rgt2snf3* double mutant (MSY441)^54^ was co-transformed the *HXT1*-*lacZ* reporter (pBM3212)^5^ with plasmids carrying the indicated *GSR* genes. β-Galactosidase activity was assayed in permeabilized cells and expressed in Miller Units. Values are means for at least three independent experiments (bottom). The *GRS* genes expressed were: *RGT2* (JKP606), *SNF3* (JKP607), *RGT2-ST* (JKP603), and *SNF3-RT* (JKP604). (**G**) The P_*HXT2*_*-hph* reporter strains (KLS75) expressing the indicated *GSR* genes were scored for growth in a SC-2% raffinose (2%)/hygromycin (200 µg/ml) plate or galactose (2%) plate (top). The plates were incubated for 2 days at 30 °C. The *rgt2snf3* double mutant (MSY441)^54^ was co-transformed the *HXT2*-*lacZ* reporter (JKP493) with plasmids carrying the indicated *GSR* genes. β-Galactosidase activity was assayed as described above (bottom).

The C-terminal cytoplasmic tails of the Rgt2 and Snf3 serve as the glucose signaling domains of the GSRs^4^ (Fig. 1A, left). Because the TM domains of Rgt2 and Snf3 generate a high- and low-glucose signal, respectively, we examine whether the Rgt2 tail can transmit the low glucose signal generated by Snf3 and vice versa. Therefore, we constructed chimeric proteins by swapping the tails of Rgt2 and Snf3—Rgt2-ST (Rgt2-TM with the Snf3 tail) and Snf3-RT (Snf3-TM with the Rgt2 tail) (Fig. 1A, right)—and studied the expression and function of the chimeric receptors. Rgt2 and Rgt2-ST protein levels are increased in cells grown on high glucose and markedly decreased when the cells are shifted to low-glucose medium (raffinose is equivalent to low glucose) (Fig. 1B,C). This is perhaps due to low glucose-induced, Rsp5-dependent endocytic degradation of Rgt2, as reported previously^6^. This is confirmed by the observation that expression of these proteins in cells lacking ubiquitin ligase activity (*rsp5-1*^*ts*^) grown at nonpermissive temperature results in increased stability (Fig. 1D,E). *SNF3* gene expression is induced by low levels of glucose (raffinose) and repressed by high glucose concentrations^5,6^. Consistently, the protein levels of Snf3 and Snf3-RT in raffinose-grown cells are higher than those in glucose-grown cells (Fig. 1B,C).

When driven from the *TEF1* promoter^22^, one of the strongest constitutive promoters, the expression pattern of *SNF3* is similar to that of *RGT2*, being maximally expressed when glucose is abundant and decreased when glucose is scarce. Moreover, Snf3 protein levels are significantly elevated in the *rsp5-1*^*ts*^ cells grown in low glucose medium (raffinose) (Fig. 1D,E). Of note, both Rgt2 and Snf3, when derived from the *TEF1* promoter, undergo endocytosis/vacuolar degradation in response to low glucose, suggesting that the receptor tails are important regulatory domains required for endocytosis and signal termination. Thus, the Rgt2 receptor is expressed only in cells grown on high glucose. By contrast, *SNF3* gene expression is increased, while Snf3 protein levels are decreasing in response to low glucose, accounting for the roles of Rgt2 and Snf3 as a high- and -low glucose receptor. Interestingly, the protein levels of Snf3-RT expressed in both glucose- and raffinose-grown cells are shown to be extremely low (Fig. 1D,E), leading us to further analyze these unexpected results in more detail in the next section below.

### The tails of the GSRs can transmit both low and high glucose signals but are not interchangeable

Next, we assessed the glucose signaling activity of the chimeric glucose receptors by determining whether their expression could restore the glucose signaling defect of a *rgt2snf3* mutant and lead to glucose induction of *HXT* expression. Because *HXT1* is induced by high glucose concentrations, the P_*HXT1*_*-hph* reporter strain grows fast on glucose/hygromycin and hardly grows on galactose^6,17^. We find that the reporter strains expressing Rgt2 or Rgt2-ST grow faster than those expressing Snf3 or Snf3-RT (Fig. 1F, top) and that expression of the wild type and the chimeric Rgt2 receptors induce a similar magnitude of P_*HXT1*_*-lacZ* expression (Fig. 1F, bottom). Similarly, expression of Snf3 and Snf3-RT is able to complement the glucose signaling defect of the P_*HXT2*_*-hph* reporter strain on raffinose (Fig. 1G, top) and results in similar levels of P_*HXT2*_*-lacZ* expression (Fig. 1G, bottom). Thus, the glucose signaling activity of the wild type and chimeric GSRs are closely correlated not with their TM or tail domains but with their expression levels.

**Figure 2:**
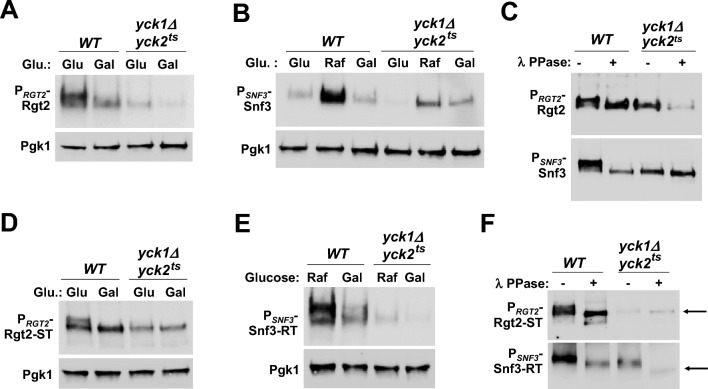
The tails of the GSRs are not interchangeable. (**A**) Western blot analysis of Rgt2-HA protein levels in *WT* (LRB939)^55^ and *yck1Δyck2*^*ts*^ (LRB1613)^55^ cells. *RGT2-HA* (JHP253) was expressed from the native promoter. Cells were grown on glucose (2%) or galactose (2%) as described in Fig. 1B, and Pgk1 was used as loading control. The *yck1Δyck2*^*ts*^ cells were incubated at 37 °C for 30 min before the precultures were shifted to fresh glucose medium or galactose medium. (**B**) Western blot analysis of Snf3-HA protein levels in *WT* and *yck1Δyck2*^*ts*^ cells. *SNF3-HA* (JKP298) was expressed from the native promoter. Cells were grown on glucose (2%), raffinose (2%), or galactose (2%) as described in Fig. 1B, and Pgk1 was used as loading control. (**C**) *WT* and *yck1Δyck2*^*ts*^ strains expressing *RGT2-HA* (JKP253) or *SNF3-HA* (JKP298) were grown on glucose as described in Fig. 1B, and whole cell lysates were immunoprecipitated with agarose-conjugated anti-HA antibody. The precipitates were treated with (+) or without (−) lambda protein phosphatase (10 U) at 30℃ for 30 min and analyzed by Western blotting with anti-HA antibody. (**D**) Western blot analysis of Rgt2-ST-HA protein levels in *WT* and *yck1Δyck2*^*ts*^ cells. *RGT2-ST-HA* (JKP602) was expressed from the *RGT2* promoter. Cells were grown as described in Fig. 1B, and Pgk1 was used as loading control. (**E**) Western blot analysis of Snf3-RT-HA protein levels in *WT* and *yck1Δyck2*^*ts*^ cells. *SNF3-RT-HA* (JKP600) was expressed from the *SNF3* promoter. Cells were grown on raffinose (2%) or galactose (2%) as described in Fig. 1B, and Pgk1 was used as loading control. (**F**) *WT* and *yck1Δyck2*^*ts*^ strains expressing *RGT2-ST-HA* (JKP602, from the *RGT2* promoter) or *SNF3-RT-HA* (JKP600, from the *SNF3* promoter) were grown on glucose as described in Fig. 1B. Whole cell lysates prepared from cells (*WT* and *yck1Δyck2*^*ts*^) grown on glucose (Rgt2-ST-HA) and on raffinose (Snf3-RT-HA), were immunoprecipitated, treated with (+) or without (−) lambda protein phosphatase, and analyzed by Western blotting with anti-HA antibody, as described above (**C**). Phosphatase treatment causes different mobility shifts of Rgt2-ST and Snf3-RT on SDS-PAGE (arrows).

It has been recently shown that the Yck-dependent phosphorylation of the tail of the Rgt2 receptor is required for its stability and function^15,17^. Snf3 also contains potential Yck consensus sites in its C-terminal domain^9^. Inactivation of the Ycks (*yck1Δyck2*^*ts*^) markedly decreases the protein levels of Rgt2 (Fig. 2A, Glucose) and Snf3 (Fig. 2B, Raffinose). Lambda phosphatase treatment causes both the Rgt2 and Snf3 receptors from the wild type cells to migrate as single bands with increased mobility in SDS-PAGE, whereas phosphatase treatment does not affect the migration of the GSRs from the *yck* mutant (Fig. 2C). These results suggest that both Rgt2 and Snf3 are phosphorylated in a Yck-dependent manner under high- and low glucose conditions, respectively, and that this phosphorylation is required for the glucose signaling.

Given that the tails of the chimeric GSRs can transduce both high and low glucose signals, we tested whether they undergo Yck-dependent phosphorylation and whether this phosphorylation could have functional implications for the chimeric GSRs. We find that Rgt2-ST (Fig. 2D, Glucose) and Snf3-RT (Fig. 2E, Raffinose) from wild-type cells migrate as two bands in SDS-PAGE and that the chimeric GSRs from galactose-grown cells migrate as one faster-migrating or enriched in the faster-migrating band of the duplet. Moreover, phosphatase treatment of the Rgt2-ST and Snf3-RT receptors extracted from the wild-type cells causes the upper bands to disappear and results in the accumulation of the lower band, indicating that the band shifts are due to phosphorylation (Fig. 2F, *WT*). Thus, the tails of the chimeric GSRs, like those of the wild type GSRs, are phosphorylated in cells grown on glucose (Rgt2-ST) or on raffinose (Snf3-RT) in a Yck-dependent manner.

Consistently, the Rgt2-ST and Snf3-RT receptors extracted from the *yck* mutant cells grown in medium with and without glucose migrate as a single band of higher mobility (Fig. 2D,E). Of note, however, phosphatase treatment slightly alters electrophoretic mobility of Rgt2-ST but causes a faster mobility of Snf3-RT (Fig. 2F, *yck1Δyck2*^*ts*^), suggesting that fusion of the Rgt2-tail to the Snf3-TM creates a potential phosphorylation site for an unknown kinase (Fig. 2F, arrows). This could influence a Yck-dependent phosphorylation of Snf3-RT and account for the extremely low levels of the chimeric receptor, when expressed from the *TEF1* promoter (Fig. 1D). Together, these results suggest that the tails of the chimeric GSRs can transmit both low and high glucose signals but are not interchangeable.

### The Ycks phosphorylate the tails of the GSRs but not the tails attached to Hxt1

**Figure 3:**
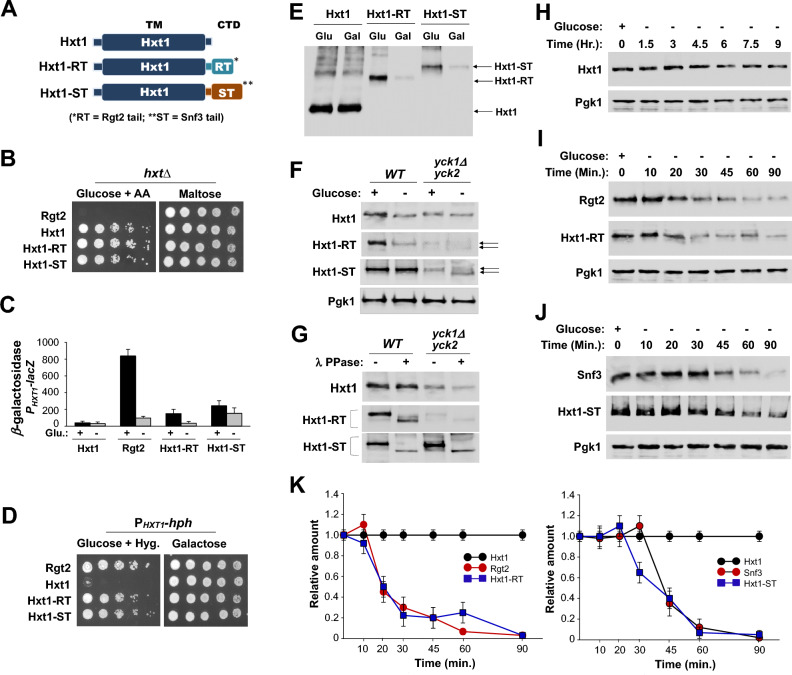
The Ycks mediate phosphorylation of the tails of the GSRs but not the tails attached to Hxt1. (**A**) Schematic representation of the structures of Hxt1 and hybrid transporter/receptor proteins Hxt1-RT (Rgt2 tail) and Hxt1-ST (Snf3 tail). (**B**) The *hxt*-null strains (EBY.VW4000, *hxtΔ*)^24^ expressing the indicated genes were scored for growth on SC-medium containing either 2% glucose with Antimycin A (1 μg/ml) or 2% maltose. The genes expressed were: *RGT2* (JKP420), *HXT1* (JKP504), *HXT1-RT* (JKP648), and *HXT-RT* (JKP650). The first spot of each row represents a count of ~ 5 × 10^7^ cell/ml, which is diluted 1:10 for each spot thereafter. The plates were incubated for 2 days at 30 °C. (**C**) The *rgt2snf3* double mutant (MSY441) was co-transformed the *HXT1*-*lacZ* reporter (pBM3212) with plasmids carrying the indicated genes. β-Galactosidase activity was assayed in permeabilized cells and expressed in Miller Units, as described in Fig. 1F. (**D**) The P_*HXT1*_*-hph* reporter strains (KLS76) expressing the indicated genes were scored for growth in a SC-2% glucose plate supplemented with 200 µg/ml hygromycin or galactose (2%) plate, as described in Fig. 1F. The plates were incubated for 2 days at 30 °C. (**E**) Expression of Hxt1 (570aa + 3HA), Hxt1-RT (788aa + 3HA), and Hxt1-ST (912aa + 3HA) was examined by Western blot analysis, and their respective genes were expressed from the *TEF1* promoter (**E**–**J**). Wild-type cells (BY4742) were grown in selective SC medium with 2% glucose to mid-log phase and equal amounts of cells were shifted to SC medium containing glucose (2%) or galactose (2%) for 1 h. Membrane fractions were immunoblotted with anti-HA antibody. (**F**) Western blot analysis of protein levels of Hxt1, Hxt1-RT, and Hxt1-ST in *WT* (LRB939) and *yck1Δyck2*^*ts*^ (LRB1613) cells. Cells were grown as described above (**E**), and Pgk1 was used as loading control. The glucose-dependent phosphorylation of Hxt1-RT and Hxt1-ST in the *yck1Δyck2*^*ts*^ strain was indicated by arrows. (**G**) *WT* and *yck1Δyck2*^*ts*^ strains expressing *HXT1, HXT1-RT,* and *HXT1-ST* were grown on glucose as described above (**E**), and whole cell lysates were immunoprecipitated, treated with (+) or without (−) lambda protein phosphatase, and analyzed by Western blotting with anti-HA antibody, as described in Fig. 1C. (**H**–**J**) Wild-type cells (BY4742) expressing the indicated genes were grown in SC-2% glucose (+) medium to mid-log phase and shifted to 2% galactose (−) medium for times as indicated. Membrane fractions were immunoblotted with anti-HA antibody, and Pgk1 was used as loading control. (**K**) The intensity of each band on the blot (**H**–**J**) was quantified by densitometric scanning, as described in Fig. 1C.

Transplantation of the GSR tails onto the Hxt1 and Hxt2 transporters converts them into a glucose receptor that generates an intracellular signal for glucose induction of *HXT* expression^4,9^, whereas expression of isolated tails leads to a constitutive glucose signaling^19^. Moreover, Rgt2 lacking its tail domain is able to generate the glucose signal if overexpressed, suggesting that the GSR tails are not required for generating the signal, but serve to enhance signaling^9,23^. However, the Yck-dependent phosphorylation of the tails is required for the GSR stability and signaling^15,17^, implicating a role of this phosphorylation in GSR-mediated glucose sensing. Therefore, we generated Hxt1 transporter-GSR tail hybrid proteins by fusing the tails of Rgt2 and Snf3 to the TM domain of Hxt1 (Fig. 3A), and expressed the resulting *HXT1-RT* and *HXT1-ST* constructs in the yeast strains EBY.VW4000 deleted for all its glucose transporters *(Δhxt1-17Δgal2*) but with functional maltose transporters^24^ and P_*HXT1*_*-hph* reporter^6^, respectively. We find that attachment of the GSR tails to Hxt1 does not inhibit the ability of Hxt1 to transport glucose (Fig. 3B) but results in only partial glucose signaling (Fig. 3C,D). Expression of Rgt2 in the *rgt2snf3* mutant results in induction of *HXT1* expression approximately 53-fold, whereas expression of *HXT1-RT* and *HXT1-ST* causes ~ 6.6-fold and ~ 13-fold induction, respectively. Thus, the Hxt1-RT and Hxt1-ST hybrid transporter/receptor proteins seem to be about 12.4% and 24.5% efficient at generating the glucose signal for induction of *HXT1* expression (Fig. 3C). Expression of *HXT1*, *HXT1-RT*, and *HXT1-ST* was confirmed by Western blot analysis (Fig. 3E).

Next, we explored a possible role of the Ycks in glucose sensing and transport by these hybrid proteins by first determining whether the stability and function of Hxt1 is regulated by the YCKs. Hxt1 from wild-type cells migrates similarly to that from *yck1Δyck2*^*ts*^ mutant cells in SDS-PAGE (Fig. 3F), and phosphatase treatment does not alter the electrophoretic mobility of the Hxt1 transporters from glucose-grown wild-type and *yck* mutant cells (Fig. 3G). These results suggest that the Ycks do not phosphorylate Hxt1. Moreover, although the GSR tails carry the Yck consensus sites, expression from the *yck1Δyck2*^*ts*^ mutant does not alter the electrophoretic mobility of the Hxt1-RT and Hxt1-ST hybrid proteins (Fig. 3F). In addition, phosphatase treatment increases the mobility of these proteins, suggesting that the Hxt1-RT and Hxt1-ST proteins may be phosphorylated independently of the Ycks (Fig. 3G). When extracted from *yck* mutant cells grown on galactose (minus (-) glucose), however, these proteins migrated as two bands, suggesting that the band shift may be due to phosphorylation, which is regulated by glucose (Fig. 3F). Thus, the Ycks have no direct role in the phosphorylation of Hxt1-RT and Hxt1-ST, which may be associated with their low signaling activity. Thus, the Ycks directly or indirectly phosphorylate the tails of the GSRs^15,17^, but not the tails of the Hxt1-RT and Hxt1-ST proteins. These results suggest that the TM domains of the GSRs are required for the Yck-dependent phosphorylation of their C-terminal tails, implicating an intramolecular interaction between the two domains.

### The Hxt1-RT and Hxt1-ST transporter/receptor hybrid proteins undergo endocytosis in a manner similar to that of the GSRs

Many yeast PM receptors and transporters are downregulated by endocytosis followed by trafficking to the vacuole for degradation. Rgt2 is targeted to and degraded in the vacuole via the endocytic pathway upon glucose starvation^6,25^. The glucose transporters Hxt1 and Hxt3 are endocytosed and degraded in a similar manner to Rgt2^26,27^. However, the protein levels of Rgt2 and Hxt1 are decreased by ~ 50% within 0.5 h^6^ and 5 h^26^, respectively, after cells grown on high glucose were shifted to glucose-depleted (galactose) medium, suggesting that Hxt1 is more stable, with a half-life exceeding several hours, than Rgt2. Our previous studies suggested that the N-terminal cytoplasmic domain (NTD) of Hxt1 is necessary for ubiquitination (K12 and K39 identified as putative ubiquitin-acceptor sites) and degradation^26^, whereas the C-terminal tails of the GSRs are required for glucose starvation-induced turnover (Fig. 1). Thus, Hxt1-RT and Hxt1-ST transporter/receptor hybrid proteins have the Hxt1 N-terminal domain (Hxt1-NTD) and the GSR C-terminal domain (GSR-CTD) at both ends. To get more insights into the endocytic regulation of glucose signaling and transport, we assessed the stability of the hybrid proteins at the plasma membrane. We find that Hxt1 remains stable on the cell surface for more than 5 h after replacing glucose in the medium by galactose (Fig. 3H), as observed previously^6^. By contrast, the protein levels of Hxt1-RT and Hxt1-ST are decreased by ~ 50% within ~ 15 min and ~ 40 min, respectively, after the glucose-grown cells were shifted to glucose-free medium (galactose) (Fig. 3I,J). Thus, the half-lives of Hxt1-RT and Hxt1-ST are similar to those of Rgt2 and Snf3 (Fig. 3K), suggesting that the hybrid proteins undergo endocytosis in a mechanism similar to that of GSRs and that they behave more like the GSRs than Hxt1 under turnover conditions.

### The Hxt1 transporter attached to the GSR tails can generate a signal in response to glucose

**Figure 4:**
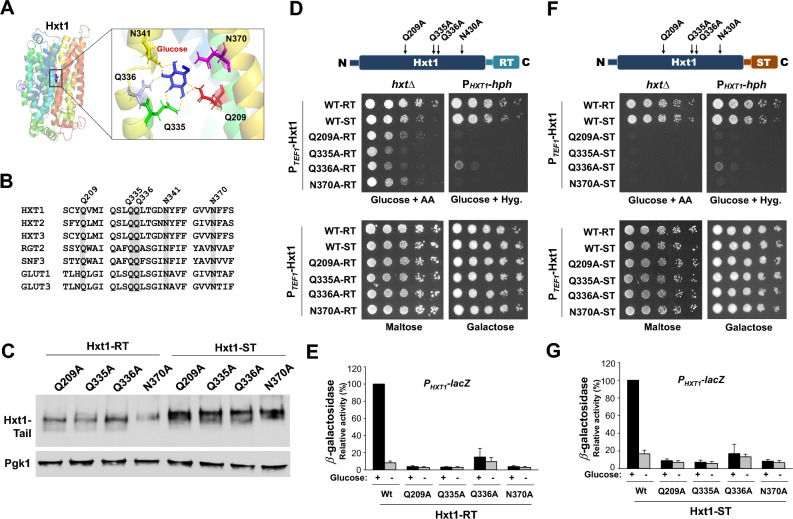
The Hxt1 TM-domain of the Hxt1-RT and Hxt1-ST hybrid proteins generates a signal in response to glucose. (**A**) Hxt1 homology model was generated using I-TASSER and visualized using PyMol, as we described previously^30^. Docking of glucose to the predicted glucose binding site in Hxt1 was visualized using AutoDock Vina^51^. (**B**) Clustal Omega was used for sequence alignment of yeast glucose transporters (Hxt1, Hxt2, and Hxt3), yeast GSRs (Rgt2 and Snf3), and human glucose transporters (GLUT1 and GLUT3). (**C**) Western blot analysis of Hxt1-RT and Hxt1-ST proteins with substitutions in residues Q209A (JKP609/JKP684), Q335A (JKP661/JKP686), Q336A (JKP663/JKP687), or N370A (JKP665/JKP 689). Yeast cells expressing the indicated *HXT1-TAIL* constructs were grown on glucose as described in Fig. 1B, and membrane fractions were immunoblotted with anti-HA antibody. Pgk1 was used as loading control. (**D**, **F**) Schematic representation of the structures of the Hxt1-RT (D) and Hxt1-ST (F) with the locations of the substitutions (top). The *hxt*-null strains (BSY.VW4000, *hxtΔ*) expressing the indicated genes were scored for growth on SC-medium containing either 2% glucose with Antimycin A (1 μg/ml) or 2% maltose. The P_*HXT1*_*-hph* reporter strains (KLS76) expressing the indicated genes were scored for growth in a SC-2% glucose plate supplemented with 200 µg/ml hygromycin or galactose (2%) plate (bottom). The plates were incubated for 2 days at 30 °C. (**E**, **G**) The *rgt2snf3* double mutant (MSY441) was co-transformed the *HXT1*-*lacZ* reporter (pBM3212) with plasmids carrying the indicated genes. β-Galactosidase activity was assayed in permeabilized cells and expressed in Miller Units, as described in Fig. 1F.

The yeast glucose transporters (Hxts) and glucose sensing receptors (Rgt2 and Snf3) belong to the sugar porter subfamily of the major facilitator superfamily (MFS) of transporters, one of the largest membrane protein families found ubiquitously in all living organisms (Fig. 4A,B)^28–30^. The putative glucose-binding residues of Hxt1—in particular Q209, Q335, Q336 and N370—are well-conserved from yeast to humans and found to be critical for glucose uptake^31^, demonstrating that Hxt1 may transport glucose in a mechanism similar to that of human glucose transporters^32^. To explore the importance of the putative glucose-binding sites for the glucose signaling activity of the Hxt1-RT and Hxt1-ST hybrid proteins, we introduced the four substitutions (Q209A, Q335A, Q336A, and N370A) into the TM domain of the *HXT1-RT* and *HXT1-ST* constructs, respectively, and confirmed their expression by Western blotting (Fig. 4C).

Overexpression of wild type *HXT1-RT* or *HXT1-ST* (from the *TEF1* promoter on a multicopy plasmid) in the *rgt2 snf3* double mutant restores the glucose signaling defect (measured with an *P*_*HXT1*_*-hph* reporter) of this mutant (Fig. 4D,F). However, this defect is not restored by overexpression of the mutant Hxt1-RT (Fig. 4D) or Hxt1-ST (Fig. 4F) proteins. These results are corroborated with the observation that overexpression of the mutant Hxt1-RTs (Fig. 4E) or Hxt1-STs (Fig. 4G) does not repair the glucose induction defect in *HXT* expression (measured with a *P*_*HXT1*_*-lacZ* reporter). Thus, the single substitutions of the putative glucose-binding residues abrogate the generation of the glucose induction signal by the TM domain of Hxt1-RT or Hxt1-ST. These results suggest that the binding of glucose to the Hxt1 TM domains of the hybrid proteins is required to generate a signal that is transmitted to the cytoplasm through their GSR C-terminal tails. Next, we analyzed glucose transport activity of the wild type and mutant versions of Hxt1-RT and Hxt1-ST in a *hxt* null strain (EBY.VW4000)^24^. Wild-type *HXT1-RT* and *HXT1-ST*, when overexpressed, are able to restore the growth defect of the *hxt* null mutant on glucose (Fig. 4D,F). However, overexpression of the mutant *HXT1-RT* and *HXT1-ST* constructs restore partially or not at all the glucose growth defect of this strain, suggesting that the tails of Rgt2 and Snf3 could affect differently the binding of glucose to the TM-domain of Hxt1.

### The Snf3 tail, when expressed independently of the TM domains, leads to glucose induction of *HXT1* expression

**Figure 5:**
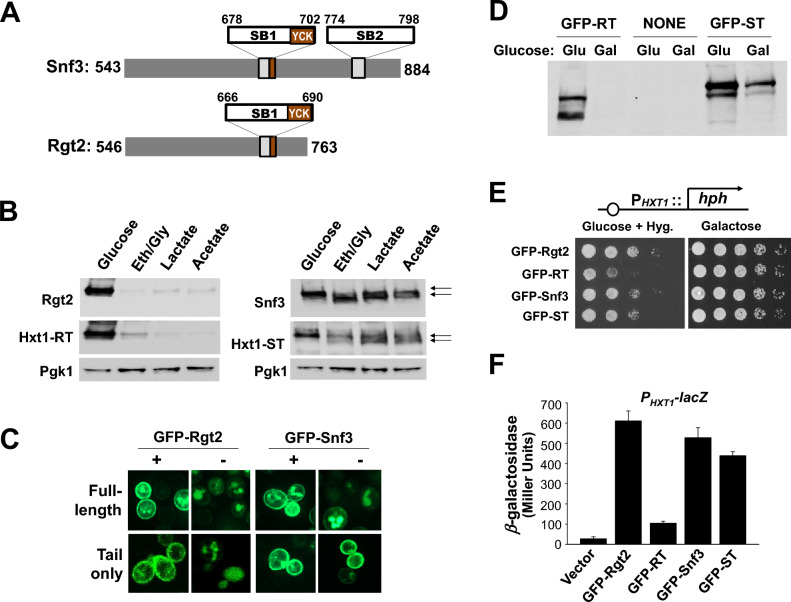
The Snf3 tail, when expressed independently of the TM domains, leads to induction of *HXT1* expression. (**A**) Schematic representation of the structures of the tails of the Rgt2 and Snf3 GSRs. The GSR tails are dissimilar, except for a signaling box (SB), a stretch of 25 amino acids that occurs twice in Snf3 and once in Rgt2. SB1 partially overlaps with the consensus sequences for the Yck phosphorylation. (**B**) Western blot analysis of protein levels of *Rgt2-HA*, *Hxt1-RT-HA*, *Snf3-HA*, and *Hxt1-ST-HA*. Wild-type cells (BY4742) were grown in selective SC medium with 2% glucose to mid-log phase (O.D_600nm_ = 1.2–1.5) and equal amounts of cells were shifted to SC medium containing glucose (2%), ethanol/glycerol (2%), lactate (2%) or acetate (1%) for 1 h. Membrane fractions were immunoblotted with anti-HA antibody. Pgk1 was used as loading control. Snf3 and Hxt1-ST from cells grown on non-fermentable carbon substrates migrate as two distinct bands on SDS-PAGE (arrows). (**C**) Confocal microscopy of wild-type (BY4742) cells expressing GFP-Rgt2 (JKP596), GFP-Rgt2 tail (JKP597), GFP-Snf3 (JKP598) or GFP-Snf3 tail (JKP616). Yeast cells were grown in glucose (2%) or galactose (2%), as described in Fig. 1B, and observed under the Zeiss LSM 510 META confocal laser scanning microscope, as we described previously^17,26^. The GFP-Rgt2 tail and GFP-Snf3-tail fragments were attached to the plasma membrane via a farnesylation signal^9^. (**D**) Expression of the tails of Rgt2 (GFP-RT, JKP597) and Snf3 (GFP-ST, JKP616) was examined by Western blot analysis. Wild-type (BY4742) cells were grown in glucose (2%) or galactose (2%), as described in Fig. 1B, and membrane fractions were immunoblotted with anti-GFP antibody. NONE: Total extracts from cells that were not transformed with a GFP plasmid. (**E**) The P_*HXT1*_*-hph* reporter strains (KLS76) expressing the indicated genes were scored for growth in a SC-2% glucose plate supplemented with 200 µg/ml hygromycin or galactose (2%) plate, as described in Fig. 1F. The plates were incubated for 2 days at 30 °C. (**F**) The *rgt2snf3* double mutant (YM6370)^48^ was co-transformed the *HXT1*-*lacZ* reporter (pBM3212) with plasmids carrying the indicated genes (pUG36 as vector). β-Galactosidase activity was assayed as described in Fig. 1F.

The GSR tails are dissimilar, except for a stretch of 25 amino acids, known as a signaling box that occurs twice in Snf3 and once in Rgt2^4,18,19^ (Fig. 5A). Early studies suggested that Snf3 transduces a signal in the complete absence of extracellular glucose (e.g., cell growth on ethanol)^21^. To get more insights into the Snf3-mediated glucose independent signaling, we first assessed the protein levels of the Snf3 receptor expressed from the *TEF1* promoter in cells grown in medium lacking glucose but containing nonfermentable carbon (NFC) sources. The Rgt2 protein levels are increased in high glucose-grown cells but markedly decreased in cells grown on glycerol/ethanol, lactate, and acetate (NFC), as reported previously^6^, whereas the Snf3 protein levels show no significant difference between cells grown on either glucose or NFC (Fig. 5B). We are surprised to discover that Rgt2 and Snf3 undergo endocytosis in response to different stimuli. More interestingly, the protein levels of *HXT1-RT* and *HXT1-ST* are very similar to those of Rgt2 and Snf3, respectively, suggesting that the tail domains contain an endocytic signal that drives endocytosis and vacuolar trafficking.

Next, we tested the ability of the GSR tails (GFP-Rgt2 tail and GFP-Snf3 tail) attached to the plasma membrane via a farnesylation signal to activate *HXT1* expression (Fig. 5C). We confirmed expression of *GFP-RT* and *GFP-ST* in cells grown on glucose (Glu) (Fig. 5D). The protein levels of GFP-RT are markedly decreased in cells grown on galactose (Gal), whereas those of GFP-ST are decreased moderately (Fig. 5D). This is perhaps due to the endocytic degradation induced by glucose starvation and is consistent with the above observations that endocytosis of the Rgt2 and Snf3 receptors may be induced by different stimuli (Fig. 5B). We find that GFP-RT and GFP-ST can restore the glucose signaling defect of the *rgt2 snf3* double mutant (Fig. 5E) and repair the glucose induction defect in *HXT1* expression (P_*HXT1*_-*lacZ*) of this mutant (Fig. 5F). Interestingly, GFP-ST is found to be more active than GFP-RT and similar to GFP-Snf3 in stimulating the high glucose-induction of *HXT1* expression, suggesting a signaling function of the isolated C-terminal tails of the Snf3 receptor.

## Discussion

The Rgt2 and Snf3 GSRs may have been derived in evolution from regular glucose transporters by acquiring a cytoplasmic tail and losing their transport activity for the purpose of glucose sensing^1,33^. We suspect that attachment of long cytoplasmic tails to Hxts renders the resulting proteins as GSRs that are conformationally unstable and non-functional, which accounts for the necessity of the Yck-dependent phosphorylation of the C-terminal tails of the GSRs^15,17^. Because the complete transport of glucose may not be required for signaling, and the structural changes occurring during the transport cycle are in some way used for signaling^33^, it is conceivable that Yck phosphorylation of the tail domains of the GSRs causes them to be arrested in a productive signaling conformation. The tail domains are required for both glucose-dependent, Yck-mediated phosphorylation to increase GSR stability at the plasma membrane and glucose starvation-induced endocytic degradation of the GSRs to stop signaling. Here, we discuss the multiple roles of the tail domains in the GSR-mediated glucose sensing and signaling.

**Figure 6:**
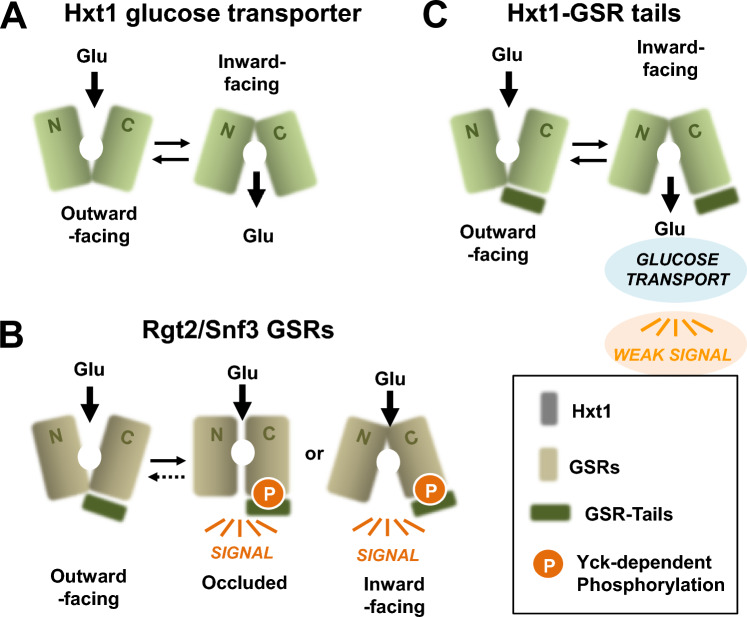
Conformation changes in glucose transporters and receptors upon glucose binding. The binding of glucose to the TM domains of the glucose transporters and receptors induces a series of conformational changes in them that are required for the transport cycle to take place^40,56^, leading to glucose transport (**A**) and glucose signaling (**B**), respectively. This difference may not be due to the long cytoplasmic C-terminal tails of the GSRs, because attachment of the tails to Hxt1 does not prevent it from transporting glucose (**C**). The considerable divergence of the TM domains of the GSRs from the Hxts probably accounts for their inability to complete the transport cycle to translocate glucose to the cytoplasmic side. Because a complete transport cycle is not required for signaling, it is believed that binding of glucose to the extracellular face of the GSRs favors their inward-facing and/or occluded form(s), which are their signaling conformation(s)^23,30^. We suggest that the Yck phosphorylation of the tail of the GSRs is required for the stabilization of the signaling conformation of the TM domain and the activation of the intracellular signaling pathway (**B**). Accordingly, the lack of the Yck phosphorylation of the tails of the hybrid proteins (Hxt1-RT and Hxt1-ST) lead to only partial signaling (**C**).

Glucose transporters use a rocker-switch alternating-access mechanism to translocate glucose across the plasma membrane, which involves a conformational alteration from an outward to an inward-facing orientation^34–36^ (Fig. 6). They are maintained in an outward-facing conformation in the absence of glucose^37,38^, and glucose binding triggers a conformational switch to an inward-facing conformation, releasing glucose into the cytoplasm^39,40^. A recent protein modeling analysis suggests that, upon glucose binding, Hxt1 undergoes a conformational change that delivers glucose into the cell, and that by contrast, the glucose-induced conformational change of Rgt2 causes it to attain a signaling conformation that activates phosphorylation of Mth1 and Std1 without the release of glucose into the cell^23^. Furthermore, constitutively signaling mutations of *RGT2* are predicted to destabilize the outward-facing form and promote the inward-facing form^23^, and the constitutively signaling Rgt2 (R231K) and Snf3 (R229K) receptors are not subjected to endocytosis and function in both the presence and absence of glucose^6^. It is thus likely that the *RGT2-1* and *SNF3-1* mutations convert the GSRs into their glucose-bound, signaling conformation. Because Yck phosphorylation of the tail domain appears to stabilize the GSRs in their signaling state (e.g., an inward-facing state), we speculate that constitutively active Rgt2-1 and Snf3-1 receptors mimic the phosphorylated state of the GSRs. This is consistent with our finding that the Hxt1-RT and Hxt1-ST hybrid proteins are able to transport glucose but retain partial signaling activity. We surmise that attachment of a long cytoplasmic C-terminal tail to Hxt1 does not prevent the Hxt1-RT/Hxt1-ST proteins from completing a transport cycle to deliver glucose into the cell, whereas the lack of the Yck phosphorylation of the tails of the hybrid proteins may hinder them from forming the signaling state that interacts with cytoplasmic signaling molecules (e.g., Mth1 and Std1 corepressors), resulting in decreased signaling. However, a more in-depth understanding of glucose transport and glucose signaling in yeast must await high-resolution crystal structures of the Hxt and GSR proteins.

The significance of the Yck phosphorylation of the GSR tail domains in glucose sensing and signaling is further underscored by our recent observations that the cytoplasmic Rgt2 receptor can generate the same signal as the cell surface Rgt2 receptor in an *akr1* mutant^17^. The Ycks are tethered to the plasma membrane through palmitoylation of the C-terminal Cys-Cys sequence by the palmitoyl transferase Akr1^41,42^. Both the Ycks and Rgt2 are co-localized to the cytoplasm in an *akr1* mutant, where the cytoplasmic Rgt2 is phosphorylated in a Yck-dependent manner and functions as an effective receptor for glucose signaling^17^. Because extracellular glucose cannot bind to the cytoplasmic Rgt2, the conformational change of the TM domain induced by glucose binding, required for the Yck phosphorylation of the tail domain, is not likely to happen in the cytoplasm. Rather, we surmise that the tail domain of the cytoplasmic Rgt2 can be directly accessible for Yck-dependent phosphorylation and that Yck phosphorylation of the tail domains is necessary and sufficient for the GSR-mediated glucose signaling.

Receptor endocytosis and degradation is an important mechanism for regulating the availability of cell surface receptors. Many yeast PM transporters and receptors are targeted to vacuole for degradation when their substrates become available in excess. Such substrate (ligand)-induced endocytic degradation of transporters may function as a regulatory mechanism to prevent excessive substrate accumulation^43^. However, endocytic degradation of the GSRs and the glucose transporters Hxt1^26^ and Hxt3^27^ is induced by glucose starvation, suggesting that the stability of glucose receptors and transporters at the plasma membrane is increased in the presence of glucose. The Hxt1-RT and Hxt1-ST hybrid proteins have short half-lives (~ 15 min and ~ 40 min, respectively), similar to those of the Rgt2 and Snf3 receptors and much shorter than that of Hxt1 (~ 5 h)^26^. The underlying mechanisms and the physiological relevance of the different half-lives of the glucose receptors and transporters are unknown. However, we cannot exclude the possibility of the binding of intracellular glucose to the cytoplasmic side of the glucose transporters (e.g., Hxt1), increasing their stability at the plasma membrane and providing for a rapid reestablishment of glucose transport when extracellular glucose is available.

The GSR signaling is primarily regulated by receptor availability at the cell surface. Rgt2 is primarily dedicated to sensing and responding to high extracellular glucose levels^5^, because it is available at the plasma membrane only when glucose is abundant. By contrast, Snf3 senses and responds to low glucose levels because its protein levels are maximal when glucose levels are low^5,6^. Because both Rgt2 and Snf3 undergo endocytosis in response to low glucose (both *SNF3* gene expression and Snf3 protein degradation are induced by low glucose), it is likely that the low glucose-triggered endocytosis of the GSRs may be induced by the same mechanism. The GSRs are also endocytosed and degraded in response to the supply of the fermentable carbon source galactose. Thus, it is generally accepted that the GSRs, like Hxts, undergo endocytosis in response to glucose depletion. More importantly, however, we find that Rgt2 is efficiently endocytosed, whereas Snf3 is endocytosis-impaired, in response to NFC substrates. This is supported by our observation that attachment of the Snf3 tail to the plasma membrane leads to glucose-induced transcription of the *HXT1* gene (Fig. 5E,F) and consistent with the previous findings that the Snf3 tail exhibits a signaling activity that is independent of the presence of extracellular glucose^19–21^. The Hxt1-RT and Hxt1-ST hybrid proteins behave like Rgt2 and Snf3, respectively, suggesting that the tail domains play an important role in GSR endocytosis. Thus, endocytosis of the Rgt2 and Snf3 receptors triggered by NFS substrates may be induced by different mechanisms. It remains unclear how this is achieved, but it might be related to the short-conserved sequence motif, a stretch of 25 amino acids in the GSR tails that occurs once in Rgt2 and twice in Snf3^3,4^. Thus, further study is warranted to determine whether this structural difference affects GSR stability and turnover.

## Methods

### Yeast strains and plasmid construction

The *Saccharomyces cerevisiae* strains used in this study were listed in Table 1. Yeast strains were grown on YP (2% bacto-peptone, 1% yeast extract) or synthetic yeast nitrogen base medium (0.17% yeast nitrogen base and 0.5% ammonium sulfate) supplemented with appropriate amino acids and carbon sources, as we described previously^6,17^. Genes were disrupted by homologous recombination using the Hygromycin or KanMX cassette^44,45^. The plasmids used in this study were listed in Table 2. The plasmids were constructed by using standard molecular biology techniques as described previously^6^. Point mutations (Q209A, Q335A, Q336A or N370A) of HXT1 were introduced by a site-directed mutagenesis kit according to manufacturer’s protocol^17^.

**Table 1:** Yeast strains used in this study.

Strain	Genotype	Source
BY4742	*MATα his3Δ1 leu2Δ0 ura3Δ0 met15Δ*	52
YM6370	*BY4742 rgt2::kanMX snf3::kanMX*	48
LRB939	*MATα his3 leu2 ura3-52*	55
LRB1613	*LRB939 yck1::KanMX yck2-2ts*	55
MSY401	*MATα ura3-52 leu2-Δ1 his3-Δ200 trp1-Δ63*	54
MSY441	*MATα ura3-52 leu2-Δ1 his3-Δ200 trp1-Δ63 snf3::hisG rgt2::HIS3*	54
KLS75	*MATα ura3-52 leu2-Δ1 his3-Δ200 trp1-Δ63 snf3::hisG rgt2::HIS3* P_*HXT2*_*-hph*	This study
KLS76	*MATα ura3-52 leu2-Δ1 his3-Δ200 trp1-Δ63 snf3::hisG rgt2::HIS3* P_*HXT1*_*-hph*	25
EBY.VW 4000	*MATa leu2-3,112 ura3-52 trp-289 his3-1 MAL2-8c SUC2 Δhxt1-17 Δgal2 Δstl1 Δagt1 Δmph2 Δmph3*	24
KFY123	*Mata his3-1 leu2-0 ura3-0 RSP5*	53
KFY124	*Mata his3-1 leu2-0 ura3-0 rsp5-1*	53

**Table 2:** Plasmids used in this study.

Plasmid	Description	Source
KFP69	pPAD80, 2μ, C-terminal 3xHA fusion	6
JKP253	P_RGT2_-RGT2-3xHA in KFP69	6
JKP298	P_SNF3_-SNF3-3xHA in KFP69	6
JKP602	RGT2-TM/SNF3-TAIL (Rgt2-ST) in JKP253	This study
JKP600	SNF3-TM/RGT2-TAIL (Snf3-RT) in JKP298	This study
JKP443	P_TEF1_-RGT2-3xHA in pRS316	This study
JKP444	P_TEF1_-SNF3-3xHA in pRS316	This study
JKP609	RGT2-TM/SNF3-TAIL (Rgt2-ST) in JKP443	This study
JKP631	SNF3-TM/RGT2-TAIL (Snf3-RT) in JKP444	This study
JKP606	JKP253 in pRS316	This study
JKP607	JKP298 in pRS316	This study
JKP603	JKP602 in pRS316	This study
JKP604	JKP600 in pRS316	This study
JKP504	P_TEF1_-HXT1-3HA in KFP69	This study
JKP648	HXT1/RGT2-TAIL (Hxt1-RT) in KFP504	This study
JKP650	HXT1/SNF3-TAIL (Hxt1-ST) in KFP504	This study
JKP659	P_TEF1_-HXT1 (Q209A)/RGT2-TAIL in KFP69	This study
JKP661	P_TEF1_-HXT1 (Q335A)/RGT2-TAIL in KFP69	This study
JKP663	P_TEF1_-HXT1 (Q336A)/RGT2-TAIL in KFP69	This study
JKP665	P_TEF1_-HXT1 (N370A)/RGT2-TAIL in KFP69	This study
JKP684	P_TEF1_-HXT1 (N209A)/SNF3-TAIL in KFP69	This study
JKP686	P_TEF1_-HXT1 (N335A)/SNF3-TAIL in KFP69	This study
JKP687	P_TEF1_-HXT1 (N336A)/SNF3-TAIL in KFP69	This study
JKP689	P_TEF1_-HXT1 (N370A)/SNF3-TAIL in KFP69	This study
JKP596	GFP-RGT2 in pUG36	This study
JKP597	GFP-RGT2-TAIL in pUG36	This study
JKP598	GFP-SNF3 in pUG36	This study
JKP616	GFP-SNF3-TAIL in pUG36	This study
JKP493	pHXT2-lacZ in yEP367R	This study

Chimeric GSRs that contain the TM domains of Rgt2 (aa 1–545) fused to the cytoplasmic tail domain of Snf3 (aa 543–884) and vice versa (Snf3 TM domains (aa 1–542) fused to Rgt2 tail domain, aa 546–763)) were constructed using gap-repair and homologous recombination. PCR-amplified overlapping DNA fragments were co-transformed with linearized KFP69 plasmid (XbaI) containing homology to both ends of the PCR products into the yeast YM6370 (*rgt2snf3*). The Hxt1 transporter (aa 1–570) fused to the cytoplasmic tail domain of Rgt2 (Hxt1-RT (aa 546–763)) or Snf3 (Hxt1-ST (aa 543–884)) was constructed in a similar manner.”

### Yeast membrane preparation

Membrane fractions were essentially prepared, as described previously^6,46^. Briefly, after washing with phosphate buffer (pH 7.4), the cell pellet was resuspended in ice cold lysis buffer (100 mM Tris–Cl, pH 8, 150 mM NaCl, 5 mM EDTA) containing protease and phosphatase inhibitors and vortexed with acid-washed glass beads. After diluting the samples with the same buffer, membrane enriched fraction was collected by centrifuging the samples at 12,000 rpm for 40 min at 4ºC. The pellet was resuspended in the lysis buffer containing 5M urea and incubated for 30 min on ice. After centrifuging at 14,000 rpm for 40 min at 4ºC, the pellet was dissolved in SDS buffer (50 mM Tris–HCl (pH, 6.8), 10% glycerol, 2% SDS, 5% β-mercaptoethanol)^13^.

### Immunoprecipitation and Western blotting

Immunoprecipitation and Western blotting were carried out as described previously^47^. Briefly, yeast cells were disrupted by vertexing with acid-washed glass beads in ice-cold RIPA buffer (50 mM Tris–HCl, pH 7.5, 140 mM NaCl, 0.1% SDS, 1% NP-40 and 0.25% sodium deoxycholate) containing protease and phosphatase inhibitors (10 mM Na-pyrophosphate, 200 mM Na-orthovanadate, 50 mM Na-fluoride). The resulting cell lysates were incubated with appropriate antibodies at 4 °C for 3 h and further incubated with protein A/G–conjugated agarose beads at 4 °C for 1 h. The agarose beads were washed three times with RIPA buffer and boiled in SDS–PAGE buffer. For lambda phosphatase treatment, the agarose beads were resuspended in 50μl of reaction buffer (100 mM NaCl, 50 mM HEPES (pH 7.5), 0.1 mM MnCl_2_, 0.1 mM EGTA, 2 mM dithiothreitol and 0.01% Brij 35) and 200U of lambda protein phosphatase for 30 min at 30C before washing again in RIPA^17^. The beads were resuspended in an equal volume of 2X SDS sample buffer, and the eluted proteins were subjected to Western blot analysis. For Western blotting, proteins were resolved by SDS-PAGE and transferred to polyvinylidene fluoride membrane (Millipore, Billerica, MA). The membranes were incubated with appropriate antibodies in TBST buffer (10 mM Tris–HCl, pH, 7.5, 150 mM NaCl, 1% Tween-20), and proteins were detected by the enhanced chemiluminescence (ECL) system^13^.

### β-Galactosidase assay

β-Galactosidase activity assays were performed using the yeast β-galactosidase assay kit (Pierce) according to the manufacturer’s instructions^48^. Results were presented in Miller Units (1000 × A_420_)/(T × V × A_600_), where A_420_ is the optical density at 420 nm, T is the incubation time in minutes, and V is the volume of cells in milliliters). The reported lacZ activities are averages of results from triplicate of usually three different transformants^48^.

### Confocal microscopy

GFP-fusion proteins expressed in yeast cells were visualized using a Zeiss LSM 510 META confocal laser scanning microscope with a 63 × Plan-Apochromat 1.4 NA Oil DIC objective lens (Zeiss)^49^. All images documenting GFP localization were acquired with the Zeiss LSM 510 software version 3.2^17^.

### Homology modeling

Clustal Omega (https://www.ebi.ac.uk/Tools/msa/clustalo/), a multiple sequence alignment program, was used for protein sequence analysis of different hexose transporters from yeast and human. The sequence of the yeast hexose (glucose) transporter 1 was obtained from Uniprot (https://www.uniprot.org)^30^. Since the crystal structure of Hxt1 is currently unavailable, we used the I-Tasser server to perform homology modeling and build the 3-dimensional structure^30,50^. 3D protein structure images were obtained using PyMol (Schrödinger, LLC). Molecular docking of glucose into the substrate binding site was performed with AutoDock Vina^51^.

### Supplementary Information

Supplementary Information.

## Data Availability

The datasets used and/or analyzed during the current study are available from the corresponding author on reasonable request.
