# Supplementary material for: Multiple roles for the cytoplasmic C-terminal domains of the yeast cell surface receptors Rgt2 and Snf3 in glucose sensing and signaling
Source: Sci Rep. 2024 Feb 19;14:4055. doi: 10.1038/s41598-024-54628-2 (PMC10876965; doi:10.1038/s41598-024-54628-2)

Fig. 1B

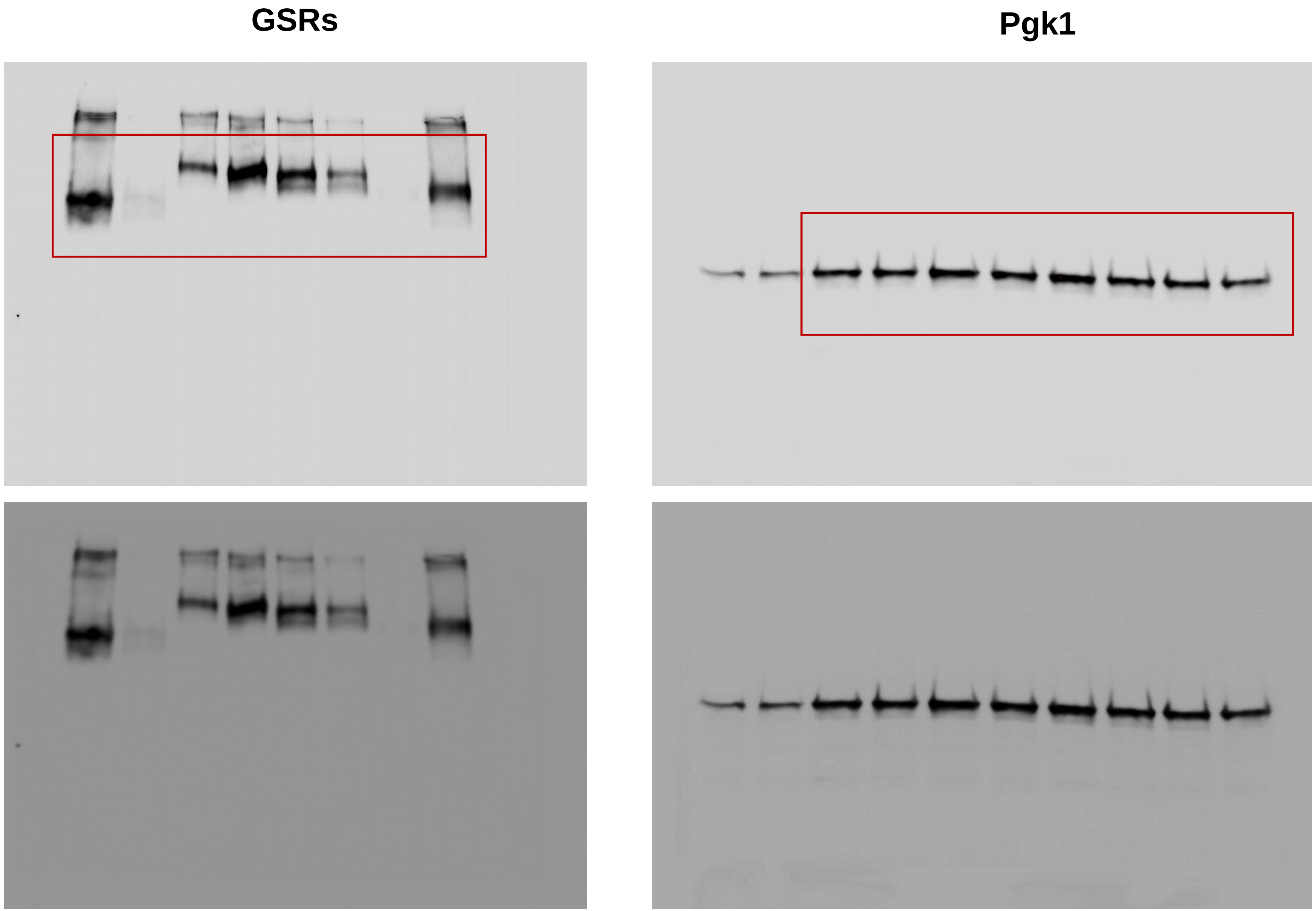

Fig. 1D

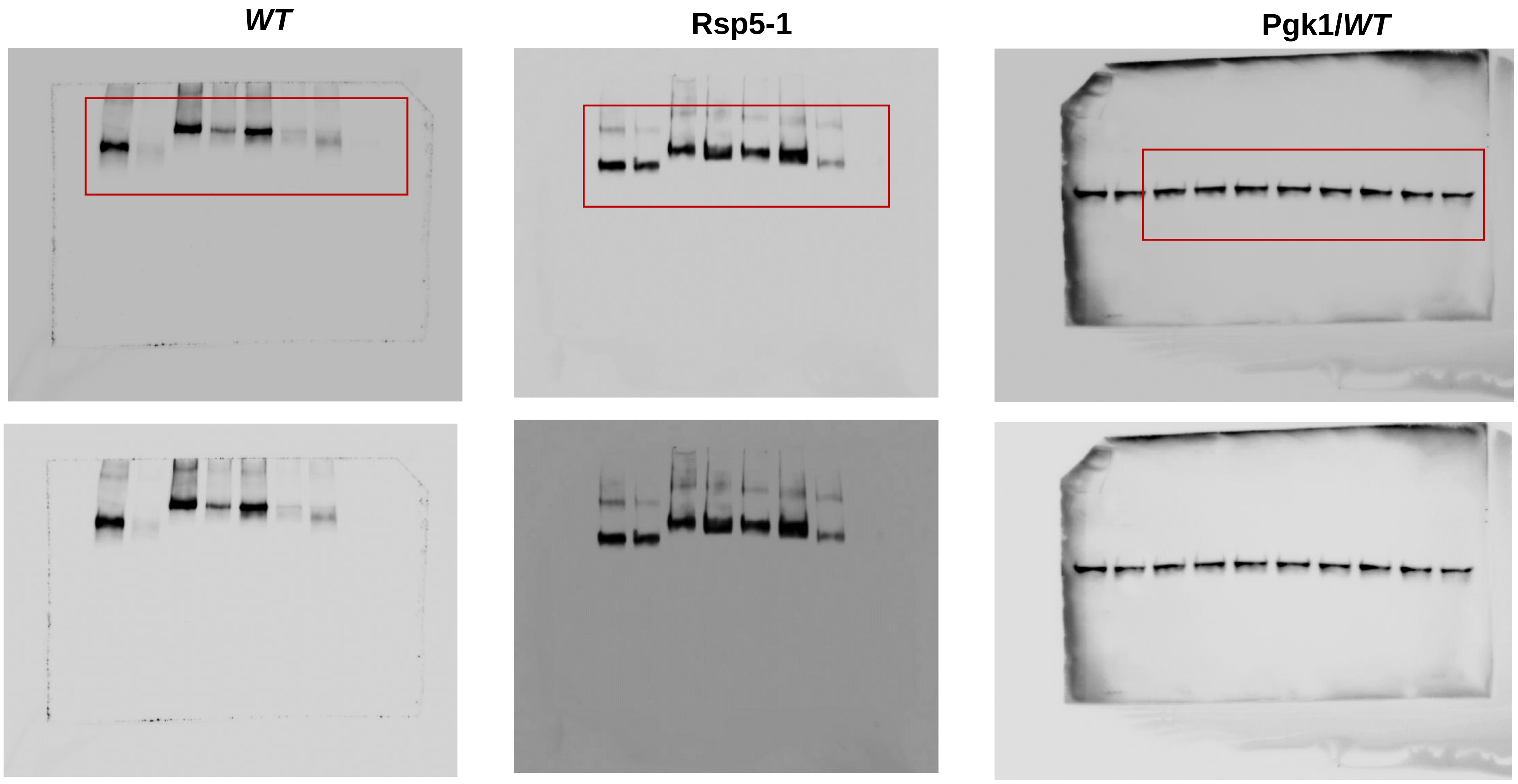

Fig. 2A

P_{RGT2}-RGT2

Pgk1

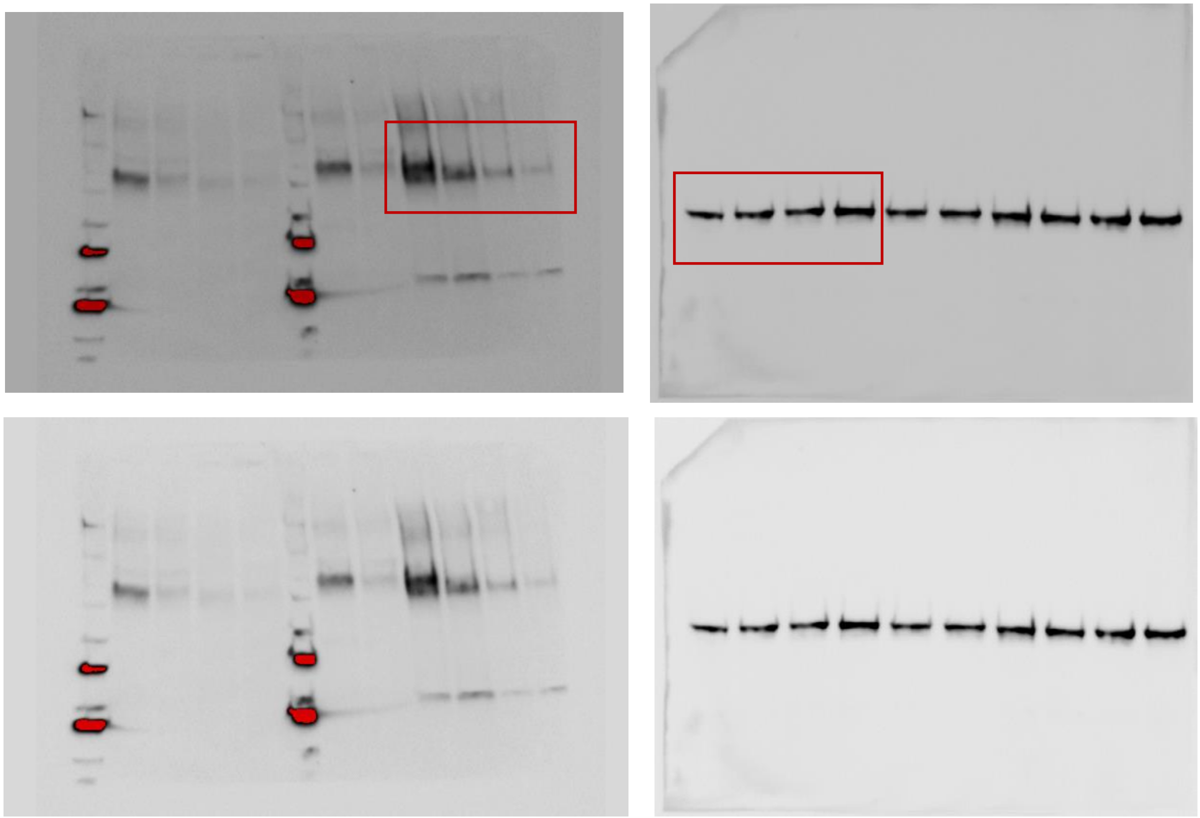

Fig. 2B

P_{SNF3}-Snf3

Pgk1

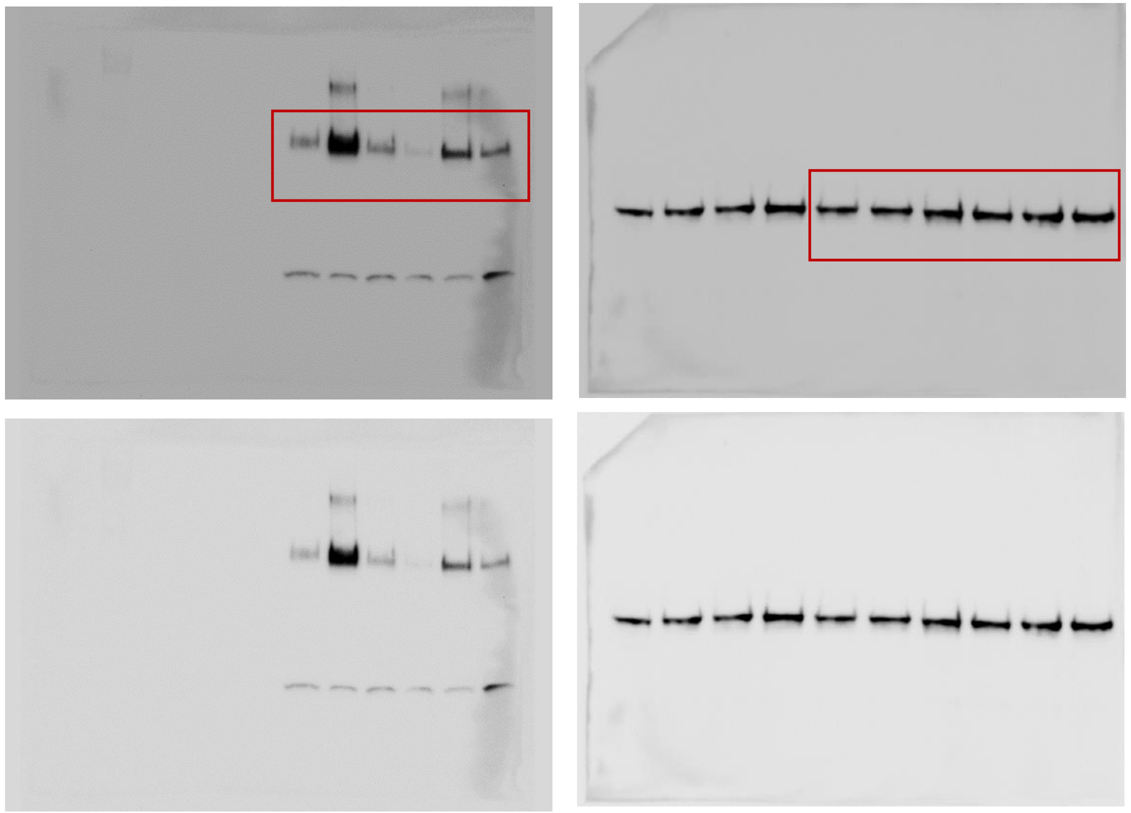

Fig. 2C

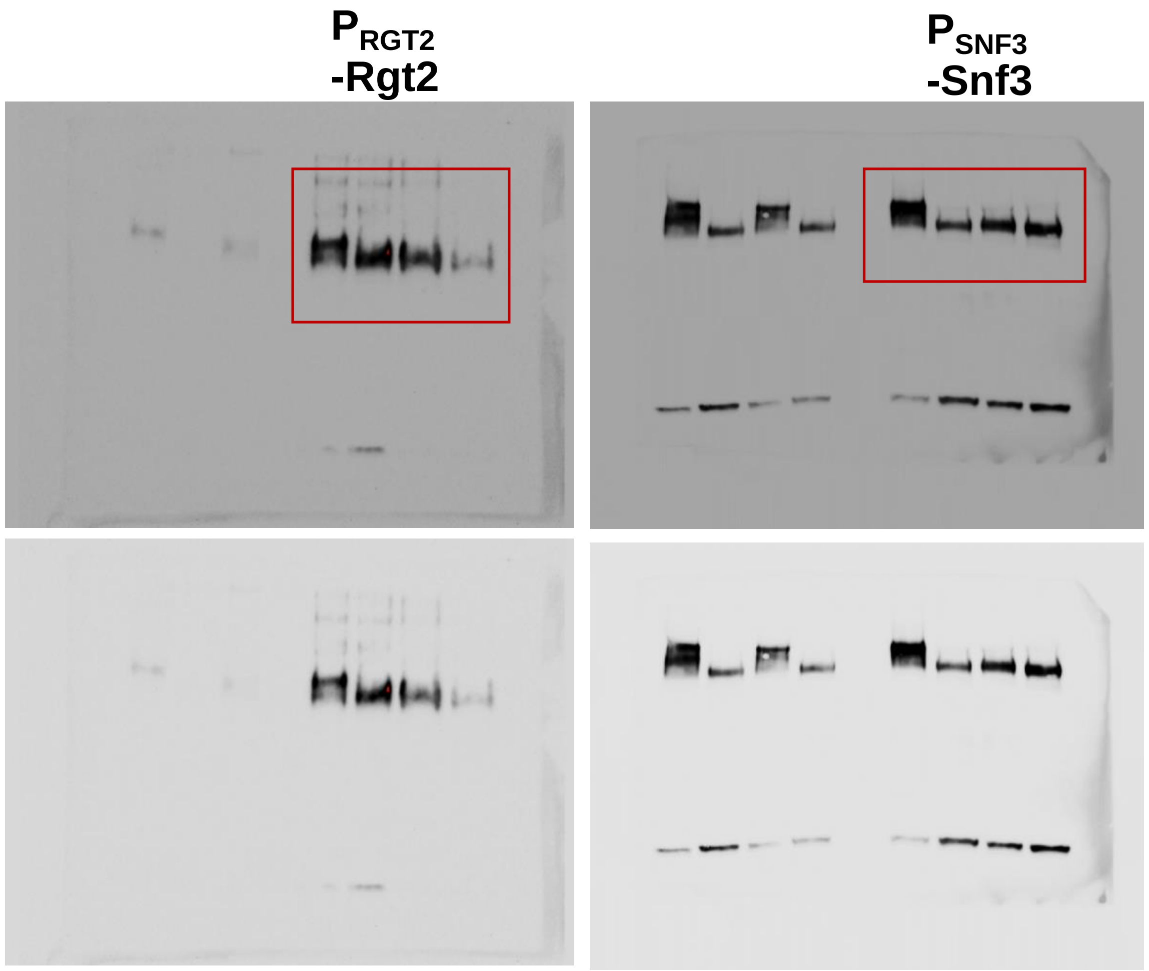

Fig. 2D

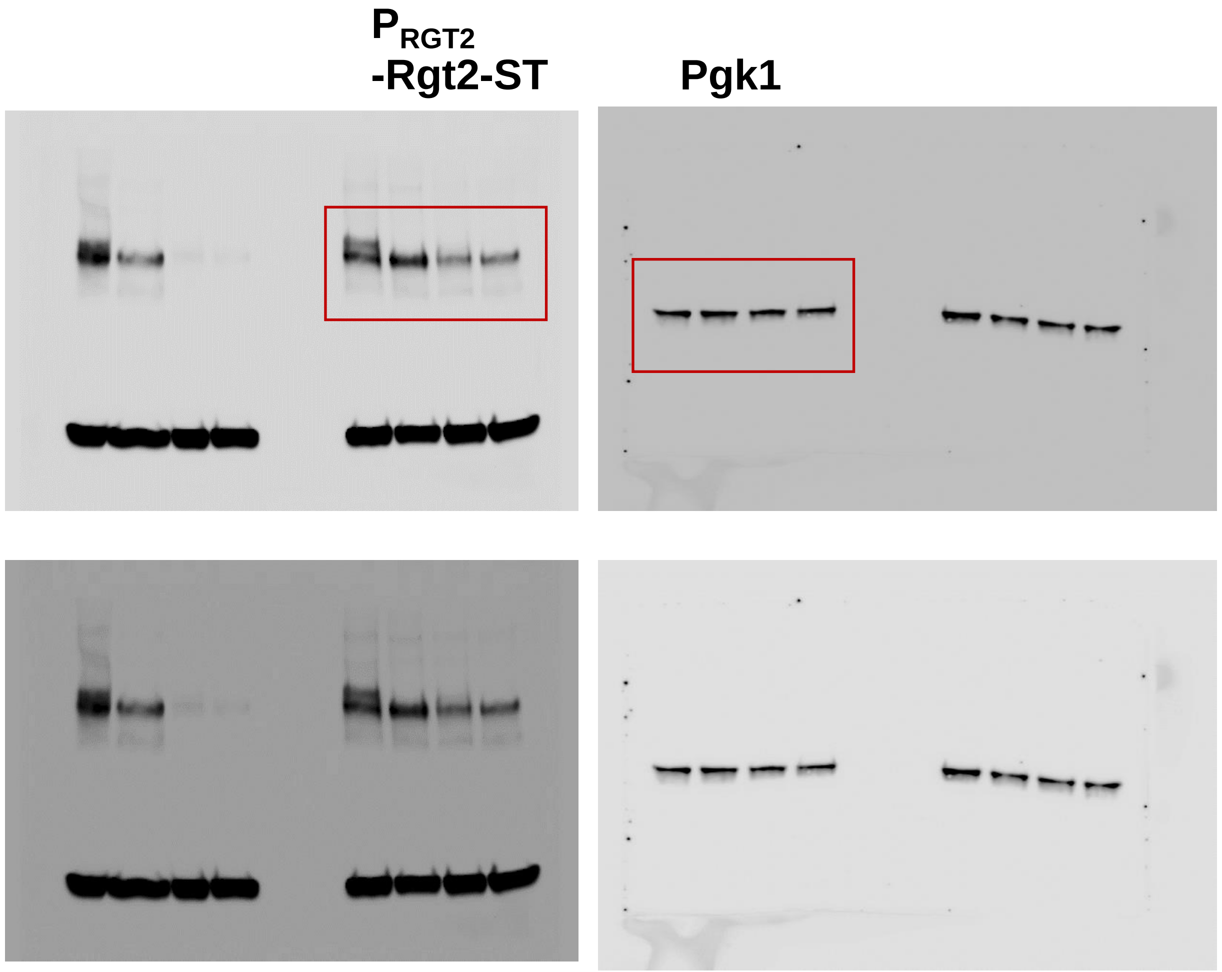

Fig. 2E

P_{SNF3}
-Snf3-RT

Pgk1

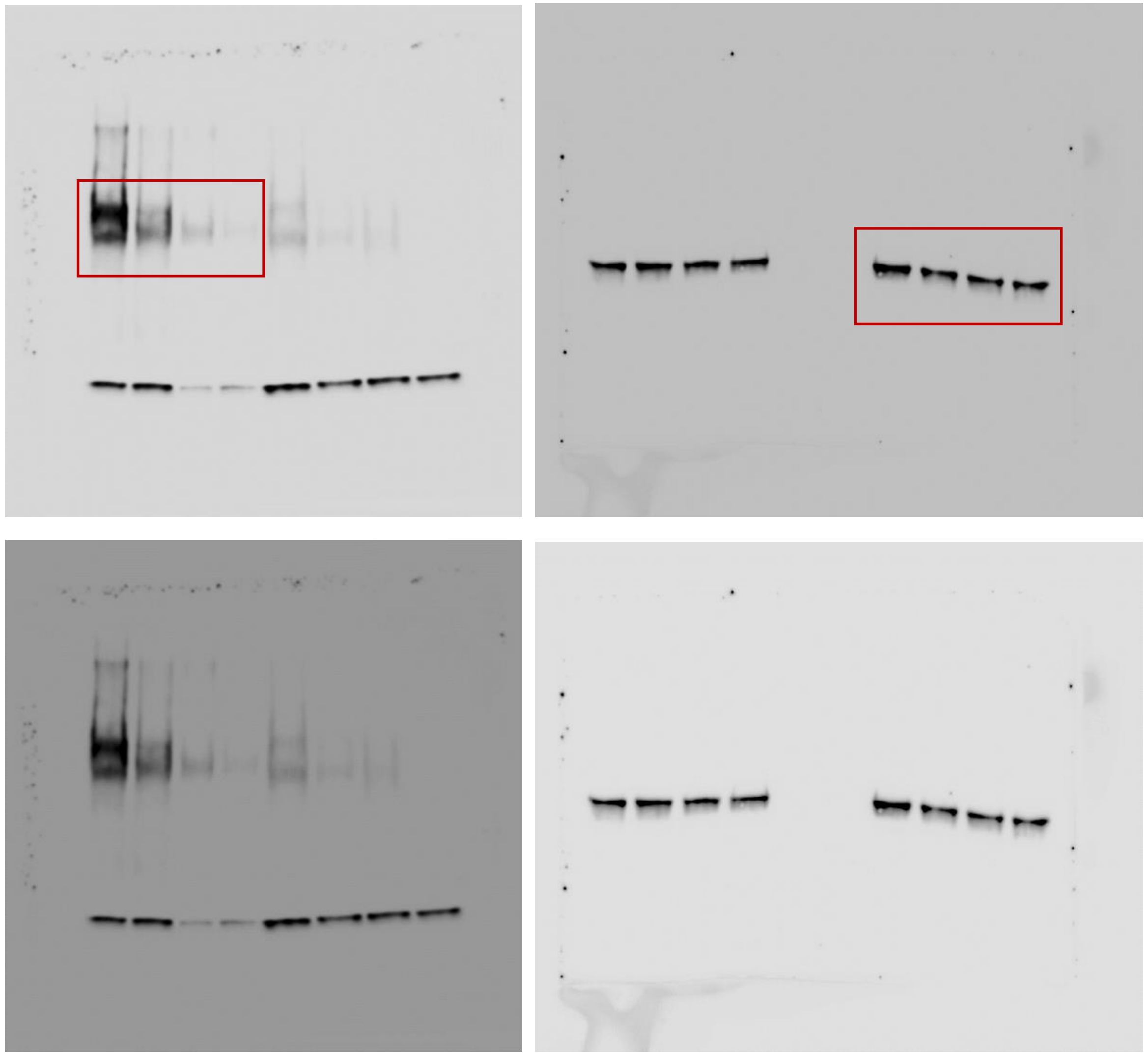

Fig. 2F

P_{RGT2}
-Rgt2-ST

P_{SNF3}
-Snf3-RT

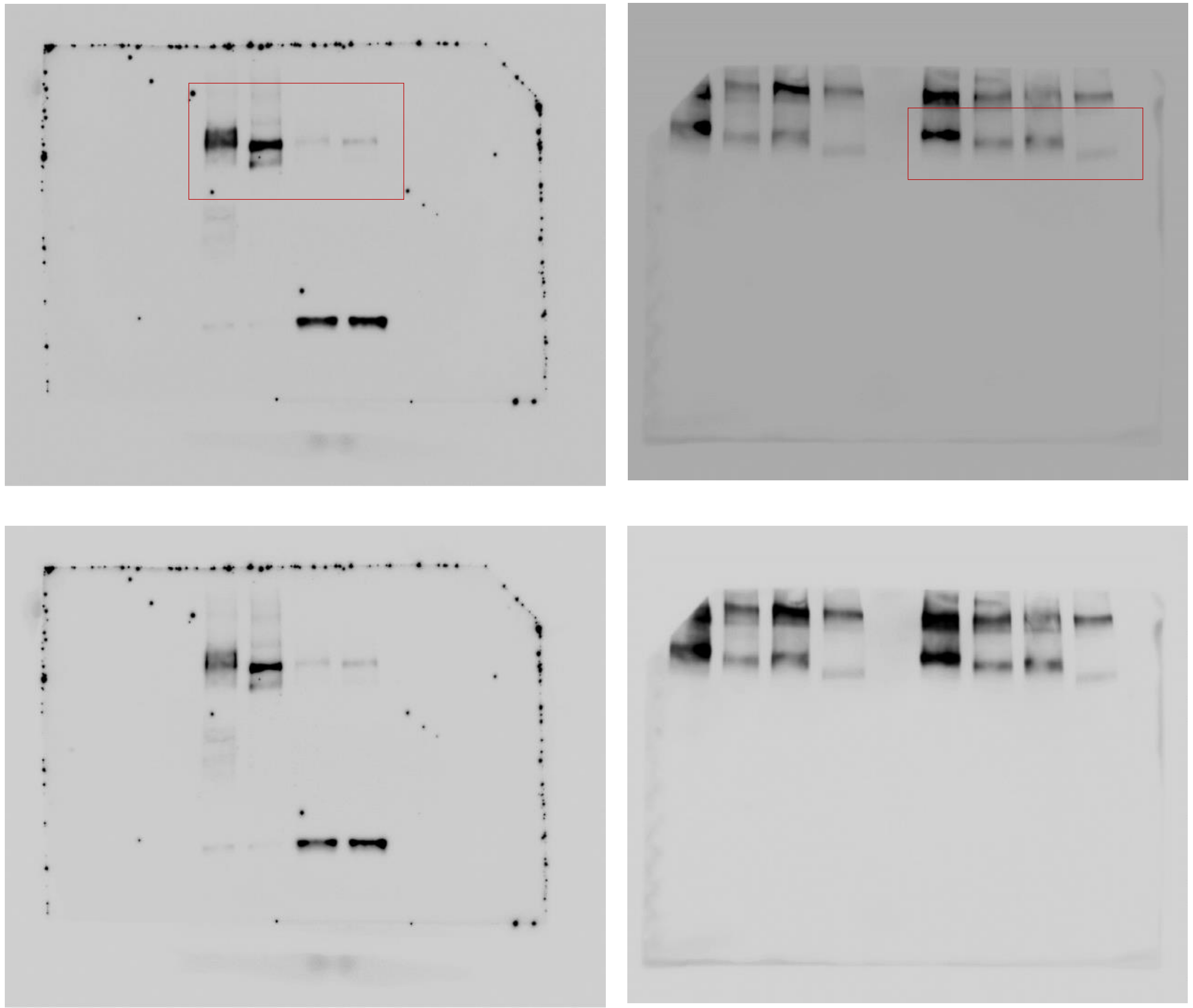

Fig. 3E

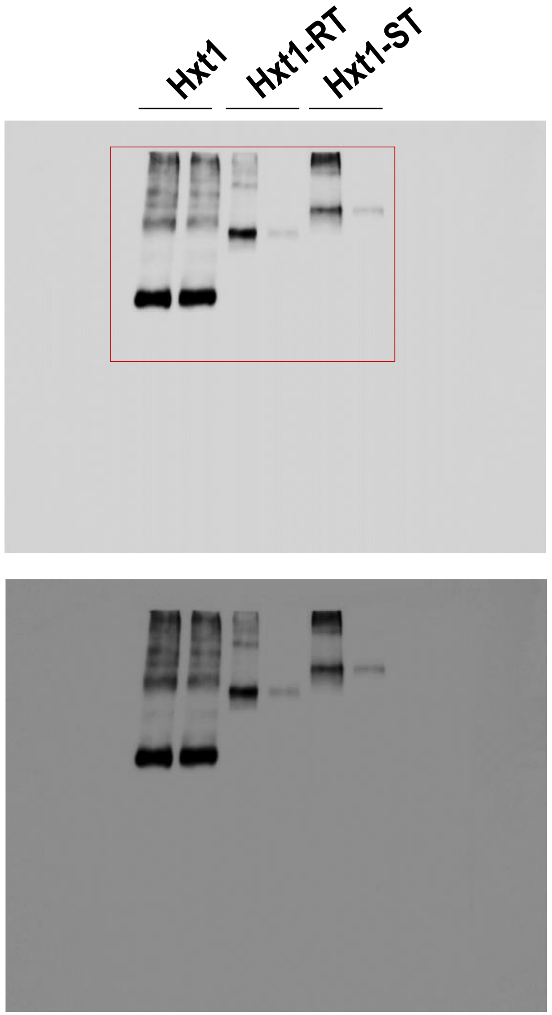

Fig. 3F

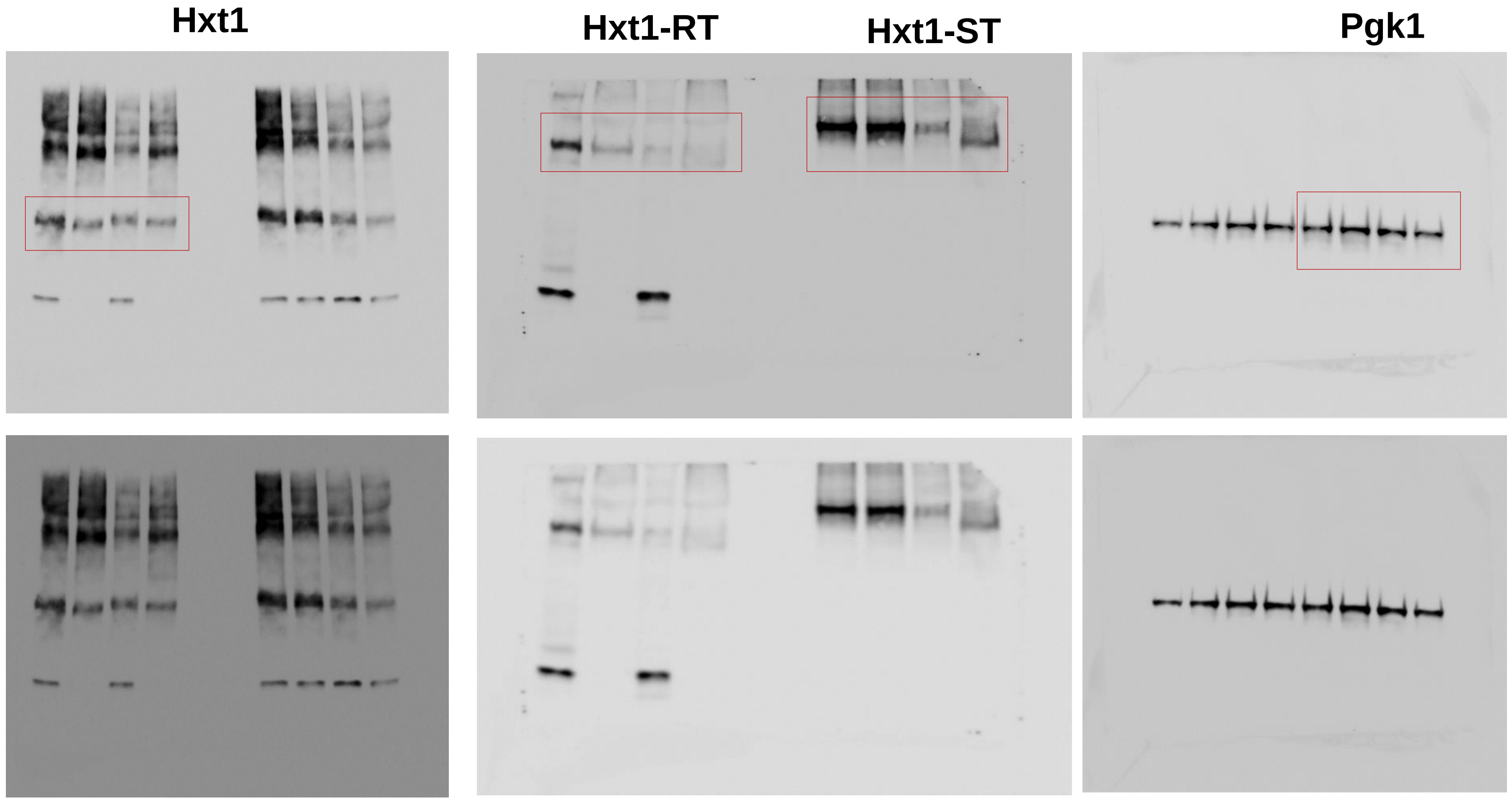

Fig. 3G

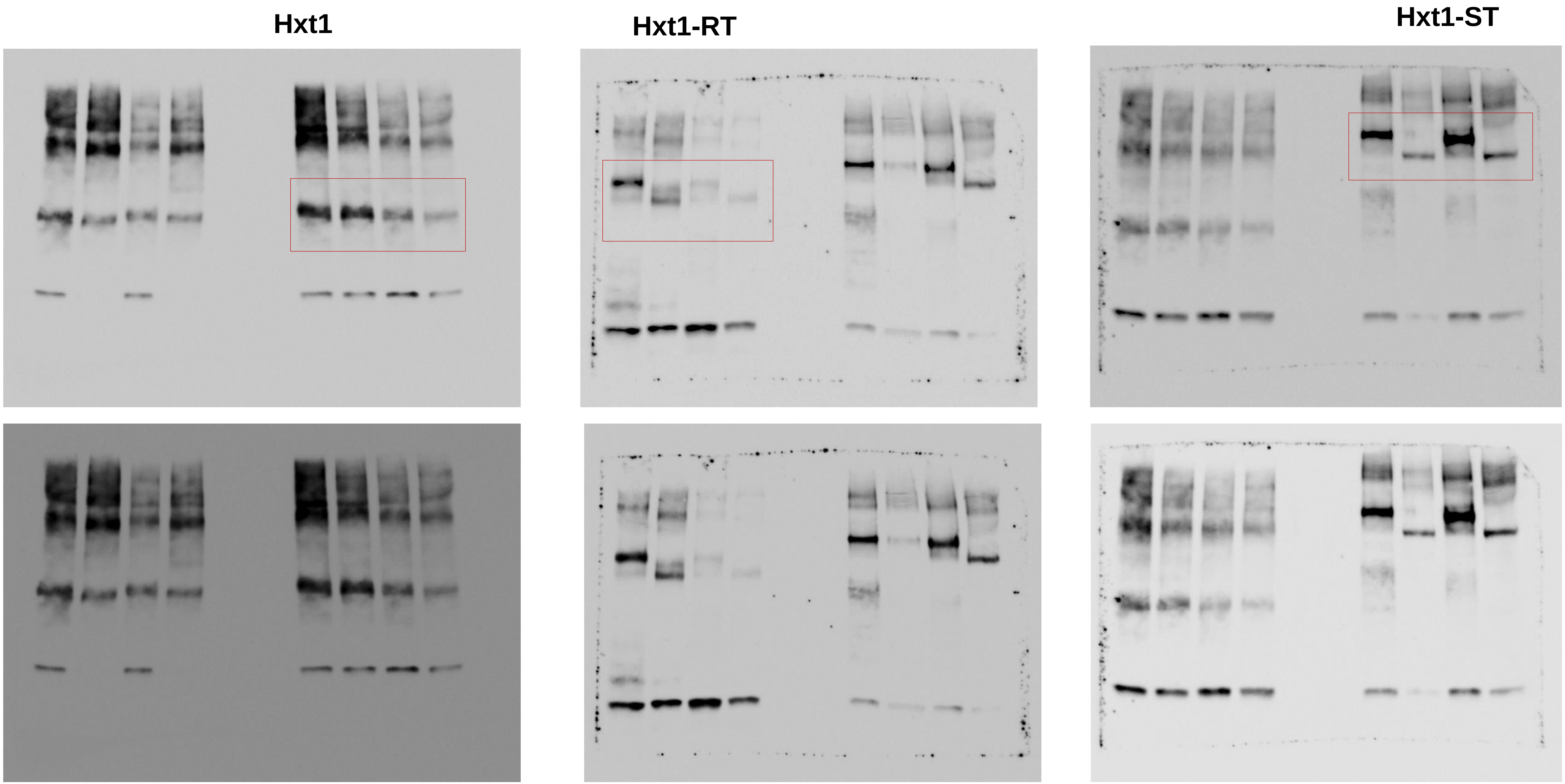

Fig. 3H

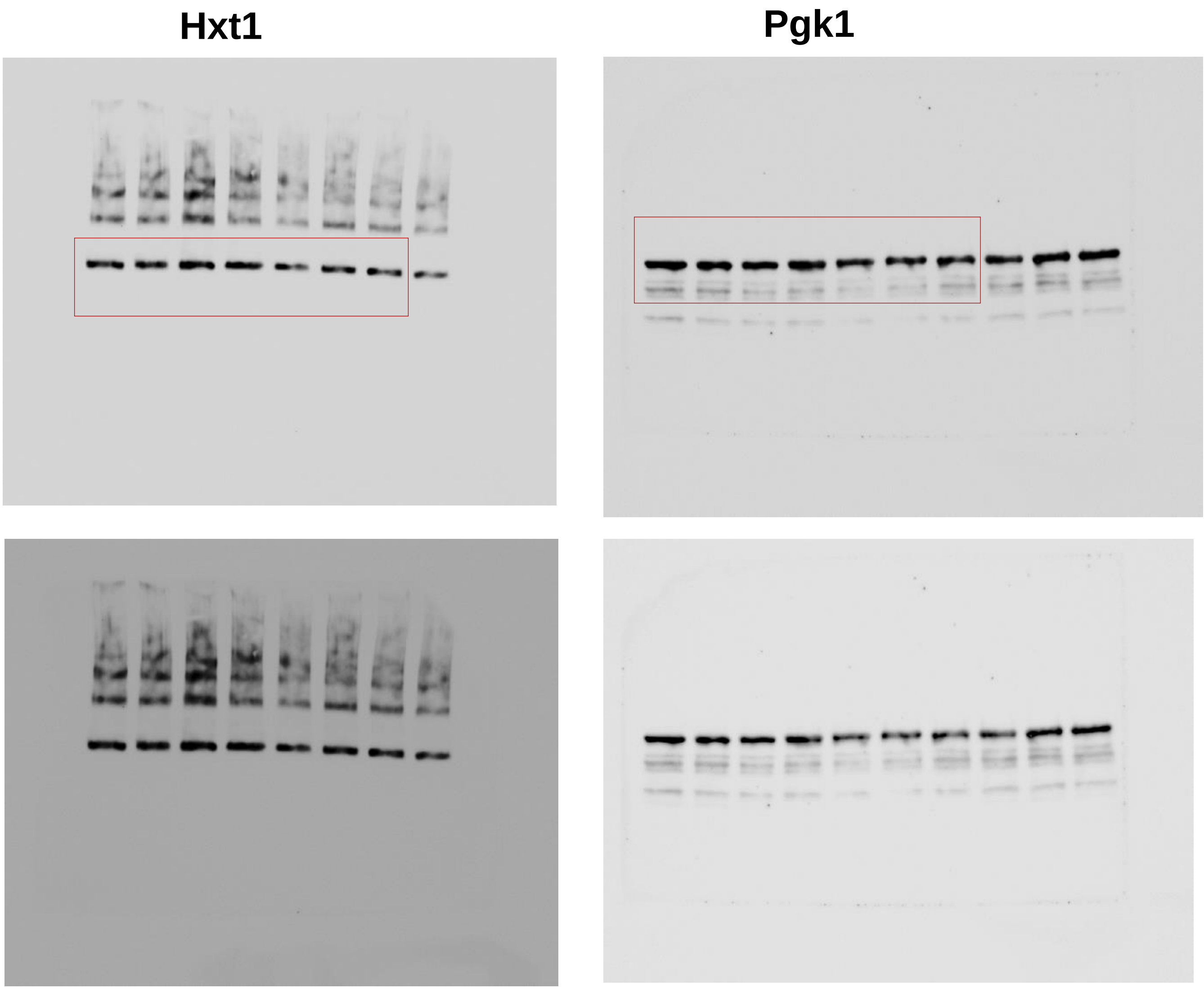

Fig. 3I

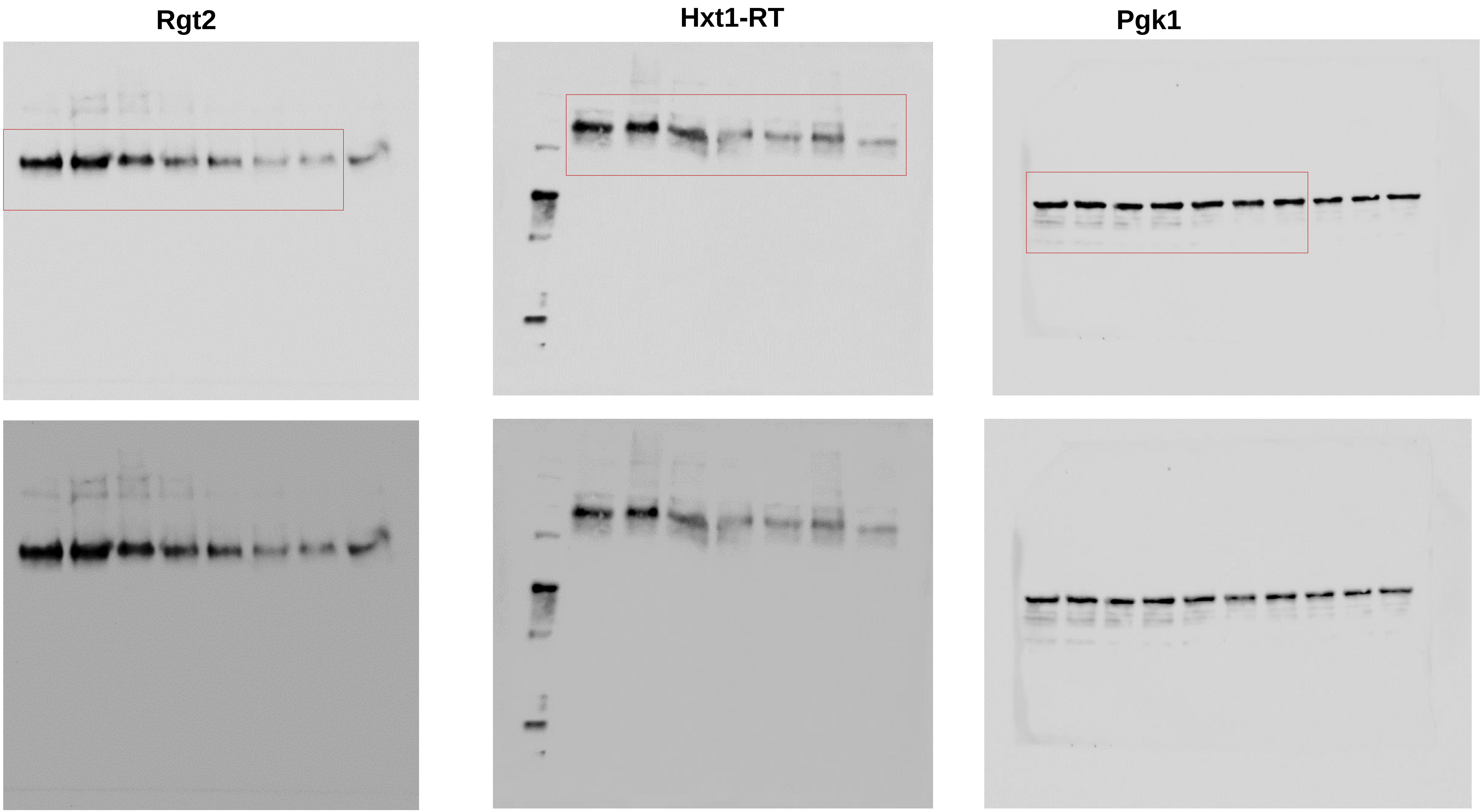

Fig. 3J

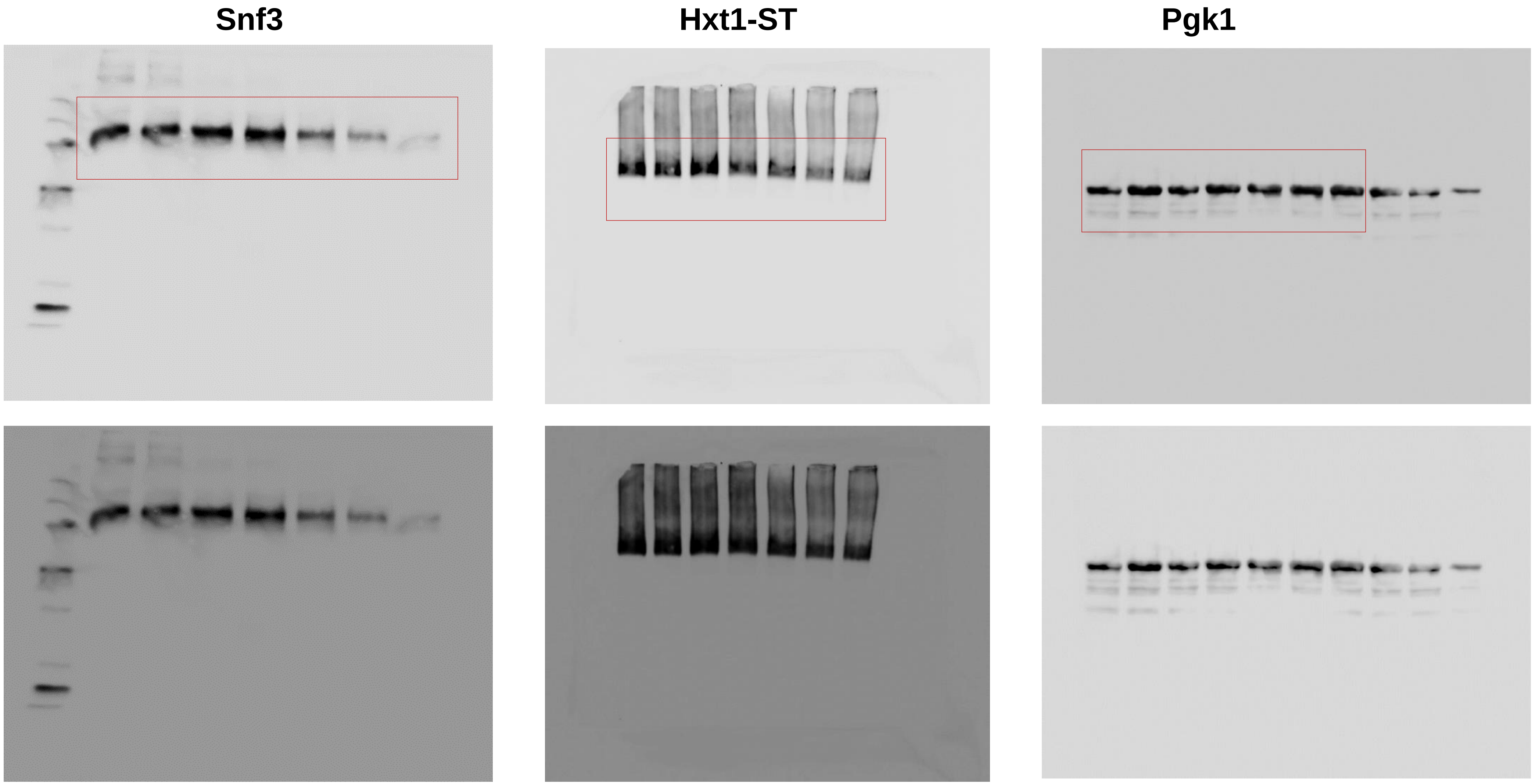

Fig. 4

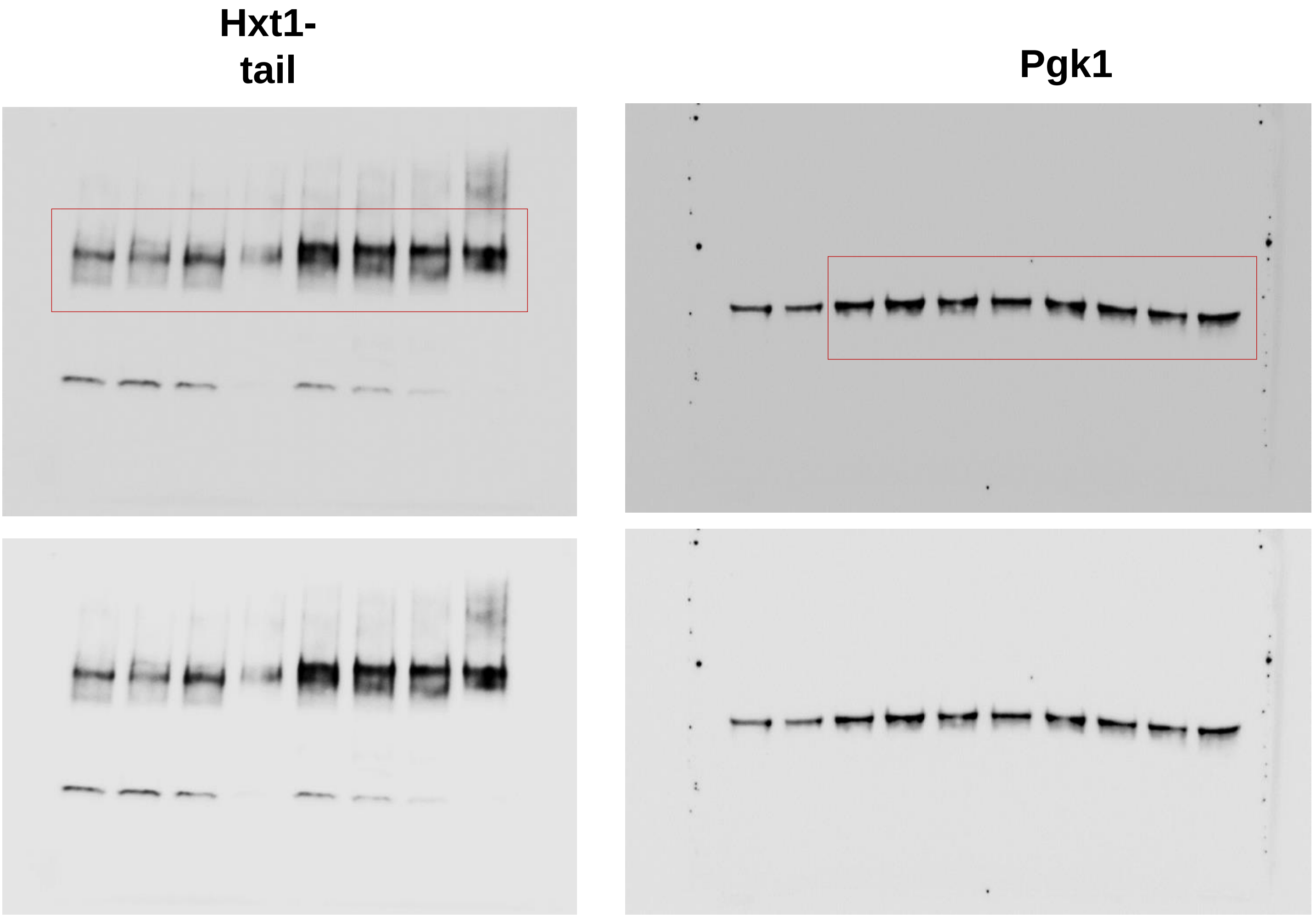

Fig. 5B

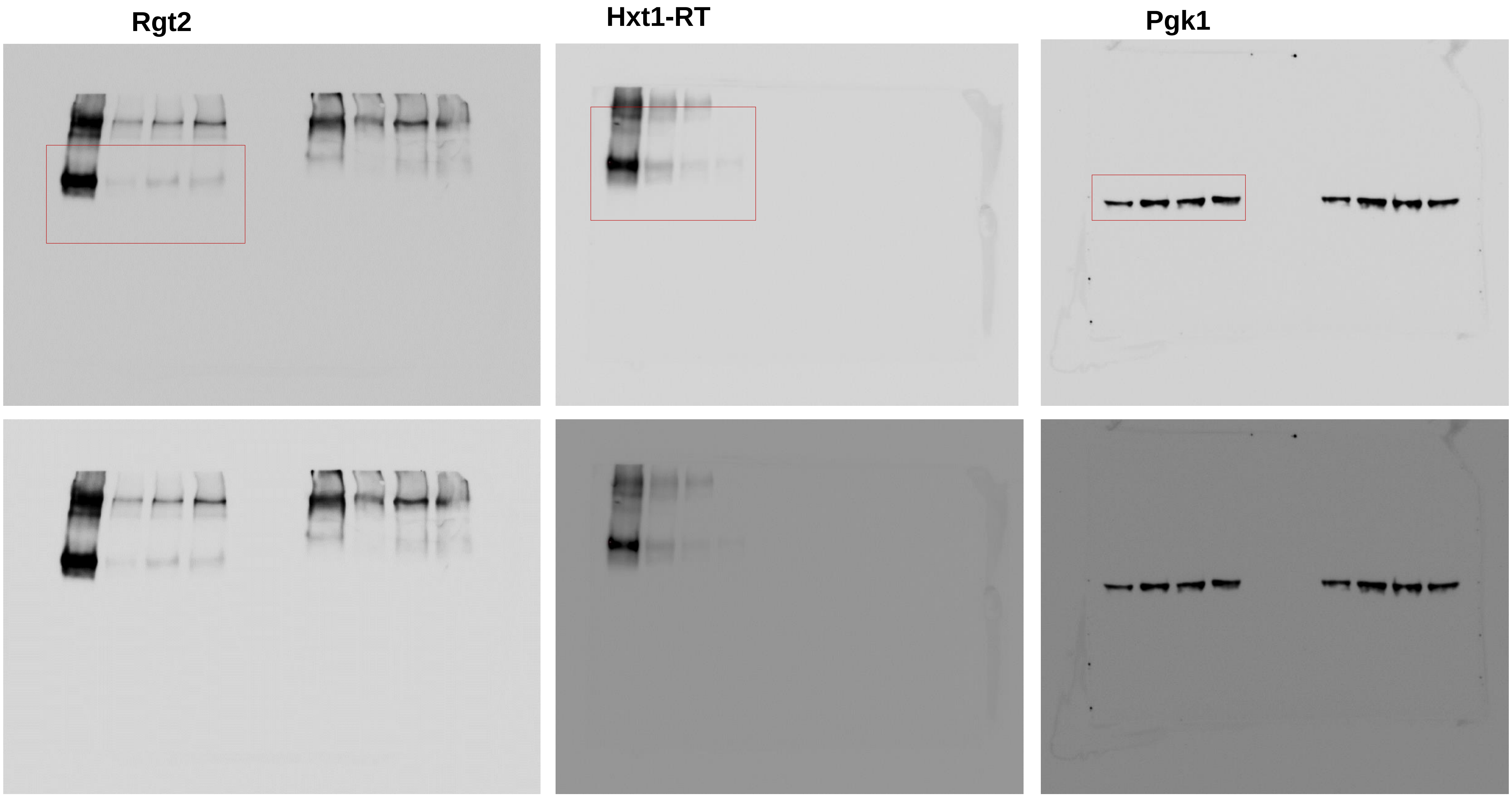

Fig. 5B

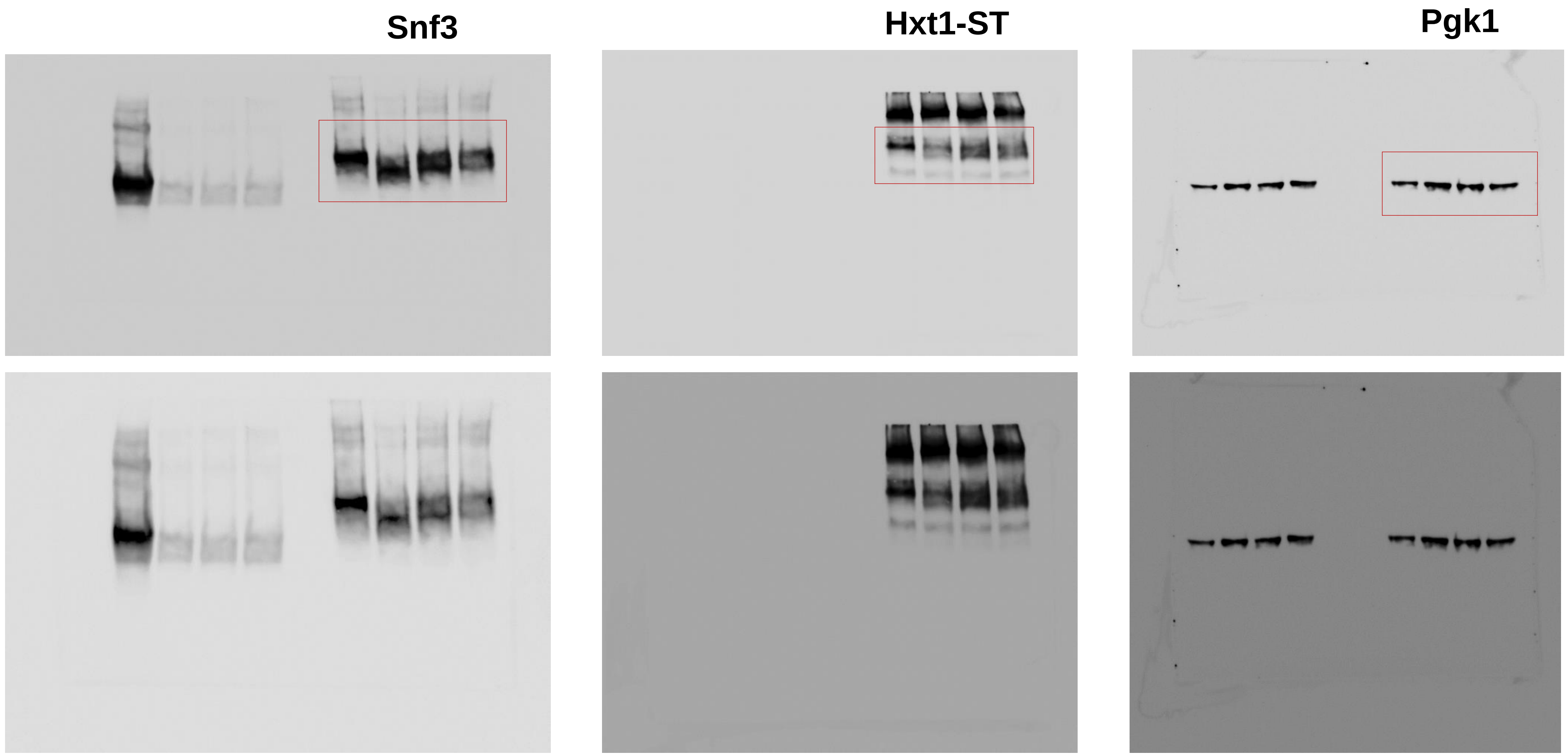

Fig. 5D

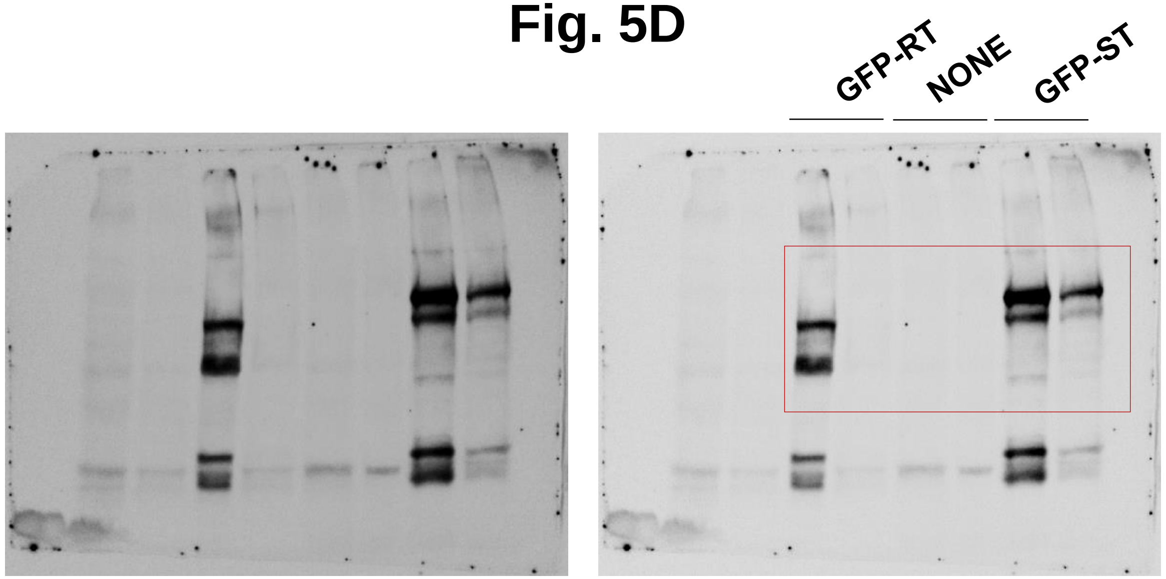

Supplement: Supplementary file 1 — Supplementary Information. [file 41598_2024_54628_MOESM1_ESM.pdf]
